# Electronic Control of Emission Behavior in Atomically Precise Copper Nanoclusters

**DOI:** 10.1021/jacsau.6c00121

**Published:** 2026-03-31

**Authors:** Maho Kamiyama, Linlin Zeng, Milan Kumar Jena, Yamato Shingyouchi, Aoi Akiyama, Tokuhisa Kawawaki, Sourav Biswas, Biswarup Pathak, Meng Zhou, Yuichi Negishi

**Affiliations:** † Institute of Multidisciplinary Research for Advanced Materials, 13101 Tohoku University, Katahira 2-1-1, Aoba-ku, Sendai 980-8577, Japan; ‡ Hefei National Research Center for Physical Sciences at the Microscale, 12652 University of Science and Technology of China, Hefei, Anhui 230026, P. R. China; § Department of Chemistry, 226957 Indian Institute of Technology Indore, Indore, Madhya Pradesh 453552, India; ∥ Department of Materials Science and Metallurgical Engineering, 486388 Indian Institute of Technology Bhilai, Bhilai, Chhattisgarh 491002, India; ⊥ Department of Applied Chemistry, 26413 Tokyo University of Science,1-3 Kagurazaka, Shinjuku-ku, Tokyo 162-8601, Japan

**Keywords:** nanocluster, copper, photoluminescence, ligands, emission

## Abstract

Atomically precise copper nanoclusters (Cu NCs) offer a compelling platform for elucidating structure–property relationships in quantum-confined materials, yet isolating ligand-induced electronic effects without altering core geometry remains a fundamental challenge. Herein, we report a systematic study of four compositionally identical Cu_11_ NCs in which the metal nuclearity and core architecture are strictly preserved, while only the substitution position and electronic nature of the thiolate ligands are varied. By employing methyl- and amino-substituted benzenethiols (ABT) in *para* and *meta* configurations, we precisely modulate the ligand-to-metal electronic communication without perturbing the Cu_11_ architecture. Despite their nearly identical atomic structures, these NCs exhibit strikingly different photoluminescence behaviors. Comprehensive steady-state and time-resolved spectroscopic analyses, complemented by transient absorption measurements and theoretical calculations, reveal that subtle changes in ligand substitution govern excited-state relaxation pathways, long-lived triplet-like excited-state stabilization, and oxygen sensitivity. Among the series, Cu_11_-3ABT achieves an exceptional photoluminescence quantum yield of 26.1% under inert conditions, arising from effective excited-state stabilization. This work establishes ligand positional engineering as a powerful and general strategy to control emission dynamics in atomically precise Cu NCs, providing fundamental insights into their excited-state physics and offering new design principles for highly emissive, earth-abundant metal NC systems.

## Graphical abstract

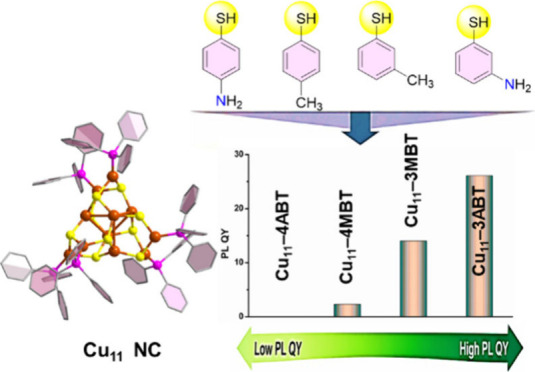

Since the emergence of atomically precise metal nanoclusters (NCs), their intriguing photoluminescence (PL) emission behavior has captured significant research attention.^1–4^ Unlike larger plasmonic nanoparticles, these ultrasmall NCs, typically comprising fewer than a few hundred metal atoms, exhibit discrete electronic states and strong quantum confinement effects.^5,6^ This quantum size regime imparts unique optical properties—especially tunable and often enhanced PL emission—that are fundamentally different from their bulk or nanoscale counterparts. Structurally, metal NCs consist of a metallic core protected by a metal–ligand shell.^7–10^ Due to their precise atomic arrangement, metal NCs display a strong structure–property correlation, wherein subtle changes in either the metal core or the ligand environment can drastically alter optical, electronic, and catalytic characteristics.^2,11–14^ Among these factors, the nature of the ligand plays a particularly decisive role.^15–18^ Beyond merely stabilizing the NCs, ligands can direct the final cluster geometry through steric, electronic, and supramolecular interactions. Consequently, rational ligand engineering has emerged as a powerful strategy to tune core architecture, surface state distribution, and ultimately modulate PL emission properties with high precision.^19–27^

Historically, research on luminescent NCs has focused predominantly on noble metals such as gold and silver, largely owing to their superior thermodynamic stability and mature synthetic accessibility.^1^ These noble metal systems have provided foundational insights into fundamental structure–property relationships in NCs. However, in recent years, copper (Cu)-based NCs have gained increasing interest—not only because of their rich photophysical behavior, but also due to natural abundance of Cu, low cost, and potential scalability for practical applications.^28–30^ Although achieving high stability in Cu­(I) NCs posed early challenges due to their susceptibility to oxidation and structural rearrangement, advancements in ligand design and synthetic methodologies have now enabled their reproducible stabilization.^31–34^

Initially, the optical properties of Cu NCs were interpreted within the framework of the jellium model, which successfully described size-dependent behavior for a range of Cu NC systems.^35^ However, as more diverse and complex structures were synthesized, notable deviations from jellium-based predictions emerged—particularly for high nuclear Cu­(I) NCs—highlighting that emission characteristics cannot be solely attributed to quantized energy levels.^28^ Instead, increasing evidence indicates that multiple factors, including ligand-to-metal charge transfer, metallophilic interactions, surface relaxation dynamics, and aggregation-induced effects, govern their PL emission behavior.

Collectively, these developments underscore the importance of understanding how atomic structure, ligand-coordination environments, and electronic configurations work together to dictate the emission mechanisms of metal NCs.^36–42^ Although several studies have investigated the emission properties of Cu NCs by varying ligand types, such changes typically alter the overall geometry of the NCs.^28,30,43–47^ Consequently, the influence of ligand structure on emission is often convoluted with geometry-induced electronic effects, making it difficult to isolate the true origin of the observed photophysical changes. To resolve this ambiguity, it is essential to design and control synthetic routes that allow systematic variation of ligand structures while preserving the underlying atomic framework of the Cu NCs.

In this work, we employ such a controlled strategy and successfully synthesize two pairs of Cu NCs that share identical metallic cores. The ligand systems were deliberately modified so that only the position of the terminal functional groups differed among the NCs, while the overall ligand backbone remained essentially unchanged. This design enables us to probe the direct influence of subtle ligand-coordination variations without altering the metal core geometry. Notably, despite their nearly identical optimized theoretical structures, the resulting NCs exhibit pronounced and well-defined shifts in their PL emission properties. These findings provide compelling evidence that even minor modifications in ligand binding orientation or electronic donation pathways can significantly modulate the emissive dynamics of Cu NCs.

Here, we successfully synthesized a series of four compositionally identical Cu NCs by systematically varying the thiolate ligands while maintaining constant nuclearity. Achieving identical metal nuclearity across ligand systems with distinct electronic environments remains a synthetic challenge; however, through careful optimization of the reaction conditions, we were able to preserve structural comparability among the NCs. To modulate the electronic environment around the thiolate coordination site, two classes of aromatic thiols were selected—methyl-substituted benzenethiols (MBT) and amino-substituted benzenethiols (ABT)—each explored in *para* and *meta* substitution patterns, yielding 4MBT, 3MBT, 4ABT, and 3ABT ligands. These substituents differ significantly in their electron-donating properties, which in turn influence the electron density at the S atom, thereby modulating the Cu–S bonding characteristics during cluster nucleation and growth. The – CH_3_ group in MBT provides a weak electron-donating effect primarily through hyperconjugation and inductive contribution, increasing electron density on the aromatic framework and subsequently at the thiolate S. In contrast, – NH_2_ in ABT acts as a considerably stronger electron-donating substituent, contributing both inductive donation and resonance effects. The lone pair on N effectively participates in π-conjugation with the aromatic ring, significantly enhancing the electron density at the S coordination center and stabilizing metal–thiolate intermediates more effectively than the methyl-substituted analogue. Furthermore, the position of substitution influences the extent of electronic communication with the thiolate group. *para*-Substituted ligands permit resonance participation, whereas *meta* substitution limits such conjugation, resulting in reduced electron donation to the thiol S. Thus, the expected trend in electron availability on the S atom follows the order: 4ABT > 3ABT > 4MBT > 3MBT. To accommodate these electronic differences during reduction-induced cluster formation, two reducing agents of different strengths were employed. A mild reducing agent, borane *tert*-butylamine complex (BTBC), was used for the methyl-substituted thiols, while sodium borohydride (NaBH_4_), a stronger reducing agent, was necessary for the amino-substituted systems due to their stronger ligand-to-metal interactions. With this controlled reduction strategy, we successfully obtained NCs of identical metal nuclearity across all ligand systems, as summarized in Scheme 1. After integration onto the NC surface, this electronic difference at the S atom is further corroborated by theoretical analysis (Table S1).

**Scheme 1:**
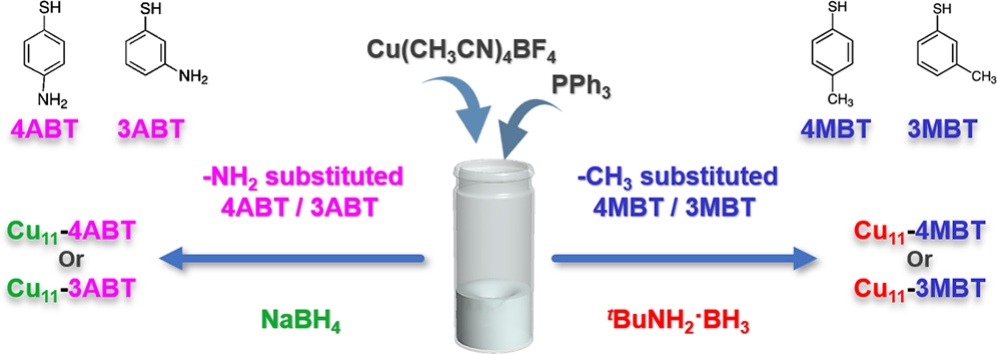
Schematic Representation of Cu_11_ NCs Synthesis with Differently Substituted Thiolate Ligands

The molecular compositions of the synthesized NCs were unequivocally confirmed by positive-mode electrospray ionization mass spectrometry (ESI-MS) analysis. All four NCs exhibited well-resolved spectral features within the *m*/*z* range of 1000–7000, as shown in Figure 1. Each spectrum displayed a single dominant molecular ion signal without fragmentation or secondary species, indicating a high degree of purity and structural uniformity across all samples. The observed *m*/*z* varied slightly among the NCs, reflecting differences in ligand structure and corresponding molecular weights. In all cases, the isotopic envelope of the parent ion exhibited a characteristic peak-to-peak separation of approximately 0.5 *m*/*z* units, confirming that the detected species carry a + 2-charge state. This charge assignment is consistent with previously reported identical [Cu_11_(4-*tert*-butylbenzenethiol)_9_(PPh_3_)_6_]^2+^ NCs.^48^ For the NCs stabilized by methyl-substituted thiols, the molecular ion peaks appeared at approximately *m*/*z* ≈ 1689 (Figure 1a-b). Comparison of the experimentally observed isotopic distribution with the simulation revealed excellent agreement, thereby confirming their molecular formulas as [Cu_11_(4MBT)_9_(PPh_3_)_6_]^2+^ (Cu_11_-4MBT) and [Cu_11_(3MBT)_9_(PPh_3_)_6_]^2+^ (Cu_11_-3MBT), respectively. Similarly, the amino-substituted thiol-protected NCs exhibited parent ion signals at *m*/*z* ≈ 1694 (Figure 1c-d). As with the methyl-substituted analogues, the simulated isotopic envelopes displayed strong overlap with the experimental spectra, validating the assigned molecular compositions of [Cu_11_(4ABT)_9_(PPh_3_)_6_]^2+^ (Cu_11_-4ABT) and [Cu_11_(3ABT)_9_(PPh_3_)_6_]^2+^ (Cu_11_-3ABT). Collectively, the mass spectrometry results confirm that all four NCs share identical nuclearity and ligand-to-metal stoichiometry, with only the positional and electronic variations of the thiolate ligands distinguishing the species. The bulk purity of these NCs was further confirmed by X-ray photoelectron spectroscopy (XPS), as the survey spectrum shows no signals corresponding to any other materials originating from the reactants (Figure S1). The high-resolution Cu 2p XPS spectra display characteristic binding energy peaks at ∼ 952.8 eV and ∼ 933.0 eV, corresponding to Cu 2p_1_/_2_ and Cu 2p_3_/_2_, respectively, unequivocally confirming the Cu­(I) oxidation state in all four NCs (Figure S2).

**Figure 1:**
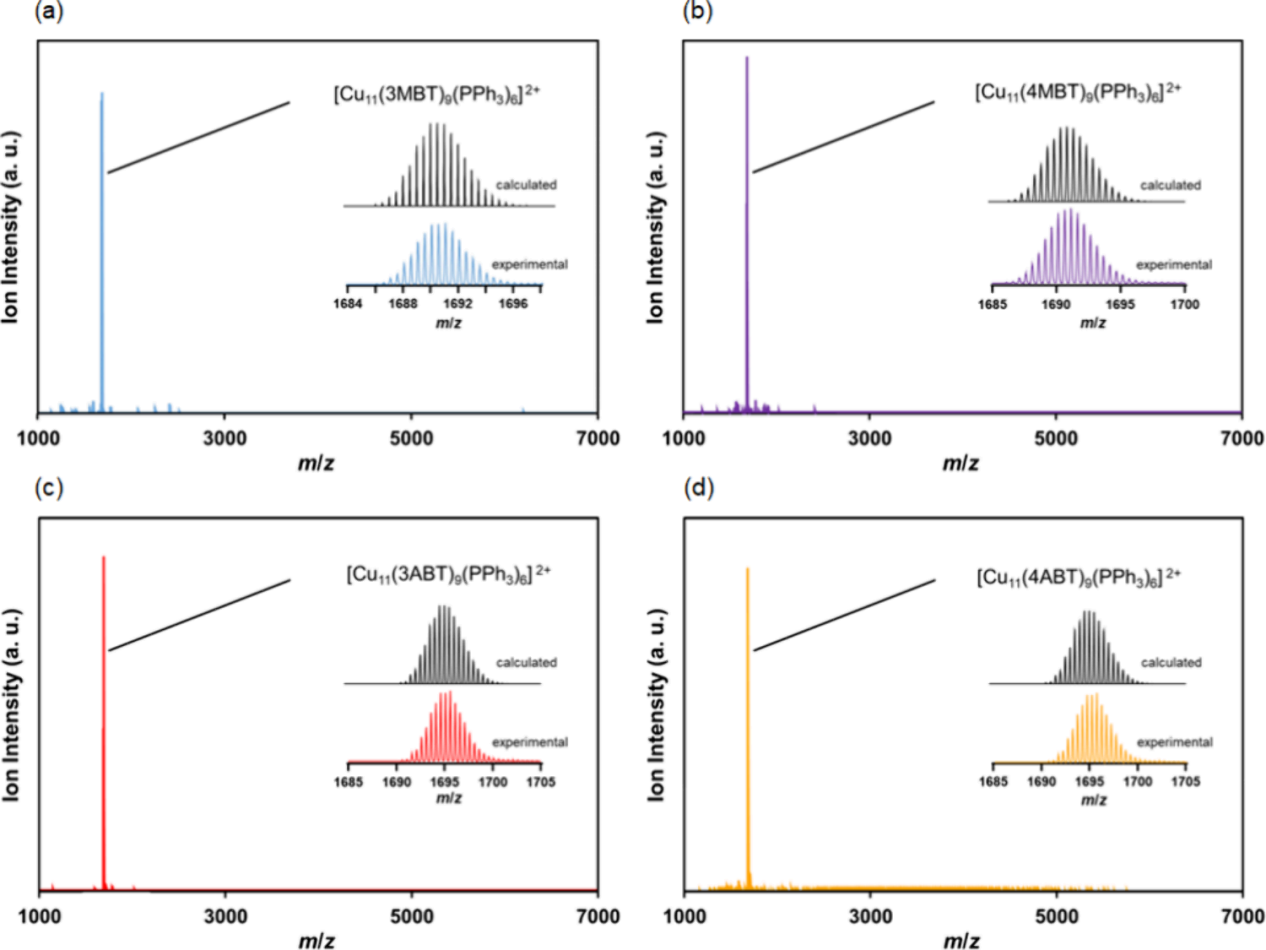
Positive-ion ESI-MS spectra of (a) Cu_11_-3MBT, (b) Cu_11_-4MBT, (c) Cu_11_-3ABT, and (d) Cu_11_-4ABT NCs are shown. The insets display the corresponding experimental–simulated spectral matches for each individual NC, confirming a + 2-charge state for all four NCs.

Single-crystal X-ray diffraction (SCXRD) analysis was attempted to elucidate the precise atomic structure of all four NCs. Among them, the crystallographic structures of Cu_11_-4MBT and Cu_11_-3ABT have been previously reported by our group, providing definitive confirmation of their core geometry, ligand arrangement, and coordination environment.^49^ These structures serve as reliable structural benchmarks for the present series, particularly given the identical nuclearity and ligand-to-metal ratio observed across all four NCs (Figure S3). However, despite extensive crystallization efforts, high-quality single crystals suitable for SCXRD could not be obtained for the remaining two NCs, Cu_11_-3MBT and Cu_11_-4ABT. Based on the previously reported SCXRD results for Cu_11_-4MBT and Cu_11_-3ABT, both NCs adopt a conserved structural motif consisting of a centrally located Cu_5_ metallic core, which is encapsulated by three Cu_2_S_3_P_2_ surface-protecting units, forming the characteristic Cu_11_ architecture. A comparative evaluation of the available SCXRD data reveals that the extent of geometric distortion or contraction within the Cu_5_ core varies as a function of the ligand electronic properties. Specifically, Cu_11_-4MBT exhibits a relatively expanded Cu_5_ core, likely due to weaker metal–ligand interactions associated with the modest electron-donating capacity of the – CH_3_ substituent. In contrast, Cu_11_-3ABT shows a more contracted core, consistent with stronger ligand-to-metal donation from the – NH_2_ group, which reinforces Cu–S interactions and stabilizes a tighter structure.

All four NCs exhibited excellent solubility in dichloromethane, enabling clear optical characterization in solution. UV–vis absorption spectra revealed a dominant feature near ∼ 405 nm for each NC (Figure 2a), suggesting that variations in ligand electronics do not significantly alter the fundamental electronic transition in Cu_11_ framework. The stability of these materials in solution was confirmed by monitoring the absorbance spectra, which showed consistent spectral profiles even after 4 h of air exposure (Figure S4). To further investigate this behavior, time-dependent density functional theory (TD-DFT) simulations were carried out on the theoretically optimized structures using the Gaussian 09 package (Figure S5).^50,51^ The average bond distances and angles of the optimized structures agree well with the experimental crystal structures (Table S2). The calculated absorption spectra showed a modest but systematic shift in the corresponding transition, with peak positions varying between ∼ 410 and 440 nm depending on the ligand identity and substitution pattern (Figure S6). Analysis of the trend revealed that NCs containing *para*-substituted ligands displayed a slight blue-shift, whereas those bearing *meta*-substituted ligands exhibited mutual red-shifted spectra. These differences correlate with variations in the HOMO–LUMO gap (Table S3) and the ligand-dependent modulation of electron density delocalization (Figures S7–S10 and Tables S4–S7). Inspection of the molecular orbital distributions further supports this interpretation (Figures S11–S14). In the *para*-substituted NCs, the occupied orbitals are predominantly localized on the metallic Cu_5_ core, with only a minor contribution from the protecting Cu_2_S_3_P_2_ units. In contrast, for the *meta*-substituted variants, the occupied orbitals exhibit increased delocalization across both the core and the ligand-derived motif shell. A similar redistribution is observed for the unoccupied orbitals (LUMO and LUMO+1), which show a greater degree of surface shell involvement in the *meta*-substituted NCs. This effect is also reflected in their natural transition orbitals (NTOs) (Tables S8–S11).^52^ These subtle perturbations in electron distribution directly contribute to the small but measurable optical shifts observed in both experimental and theoretical spectra.

**Figure 2:**
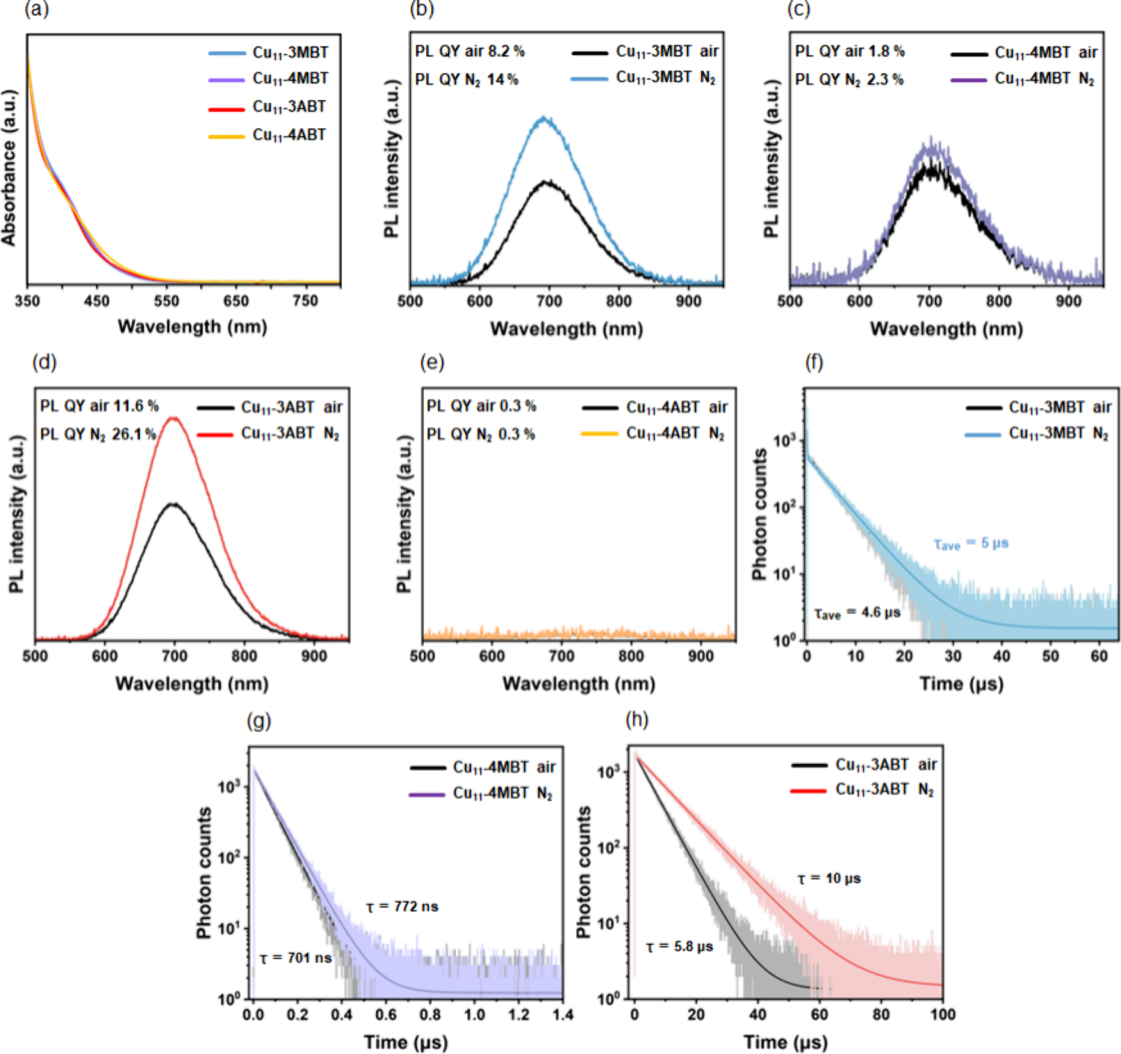
(a) UV–vis absorption spectra of all four Cu_11_ NCs recorded in in dichloromethane at room temperature under ambient condition. Room-temperature PL emission spectra of the individual NCs measured in in dichloromethane under ambient air and N_2_ atmospheres for (b) Cu_11_-3MBT, (c) Cu_11_-4MBT, (d) Cu_11_-3ABT, and (e) Cu_11_-4ABT NCs. TCSPC lifetime decay profiles collected at room temperature under air and N_2_ atmospheres for (f) Cu_11_-3MBT, (g) Cu_11_-4MBT, and (h) Cu_11_-3ABT NCs.

PL measurements provided additional insight into how ligand electronics influence excited-state behavior. Upon excitation at 400 nm, three of the four NCs (Cu_11_-3MBT, Cu_11_-4MBT, and Cu_11_-3ABT) exhibited emission maxima near ∼ 700 nm, but with markedly different intensities (Figures 2b-d). In contrast, Cu_11_-4ABT exhibits a very weak and poorly resolved emission profile, making it difficult to unambiguously determine the exact emission maximum (Figure 2e). Quantification of photoluminescence quantum yield (PL QY) confirmed this trend, with values of 0.3% for Cu_11_-4ABT, 1.8% for Cu_11_-4MBT, 8.2% for Cu_11_-3MBT, and 11.6% for Cu_11_-3ABT.

To determine whether emission behavior was influenced by surface-mediated nonradiative quenching pathways, PL measurements were repeated under a nitrogen (N_2_) atmosphere. It should be noted that N_2_ purging can significantly reduce dissolved oxygen but does not necessarily lead to strictly oxygen-free conditions. While *para*-substituted NCs showed minimal changes (Cu_11_-4ABT remained unchanged; Cu_11_-4MBT increased only marginally from 1.8% to 2.3%), a substantial enhancement was observed for the *meta*-substituted analogues (Figures 2b-e). Specifically, the PL QY increased from 8.2% to 14.0% for Cu_11_-3MBT and from 11.6% to 26.1% for Cu_11_-3ABT under N_2_ conditions. This dramatic improvement indicates a strong dependence of excited-state stabilization and oxygen-mediated quenching on the ligand substitution pattern and associated electronic structure.

To gain deeper mechanistic insight, we performed time-correlated single-photon counting (TCSPC) measurements under both oxygenated and deoxygenated conditions. All three NCs showed measurable emission lifetime shifts upon oxygen removal, indicating the involvement of oxygen-sensitive excited states (Figures 2f-h). For Cu_11_-4MBT, the emission lifetime increased modestly from 701 to 772 ns, whereas both *meta*-substituted systems exhibited significantly longer microsecond-scale emission lifetimes. The average emission lifetime of Cu_11_-3MBT increased from 4.6 to 5.0 μs, with biexponential decay fits showing a minor short-lived component (<2%). While, Cu_11_-3ABT showed an enhancement in emission lifetime from 5.8 to 10 μs upon deoxygenation, suggesting a stronger sensitivity of its excited state to the local environment. Overall, the emission lifetime variation primarily reflects modulation of the dominant long-lived excited state rather than the emergence of a new emissive pathway.

To further elucidate the origin of the PL emission, we investigated the excited-state dynamics using nanosecond transient absorption (ns-TA) spectroscopy under ambient air (Figure 3). The ns-TA measurements confirmed the TCSPC trend, with both *meta*-substituted NCs exhibiting long-lived excited states (5.3 μs for Cu_11_-3MBT and 4.8 μs for Cu_11_-3ABT), supporting the assignment of phosphorescence-like emission. In comparison, Cu_11_-4MBT displayed a shorter lifetime of 670 ns, and Cu_11_-4ABT showed an even weaker excited-state persistence with only 24 ns, suggesting a rapid nonradiative decay.

**Figure 3:**
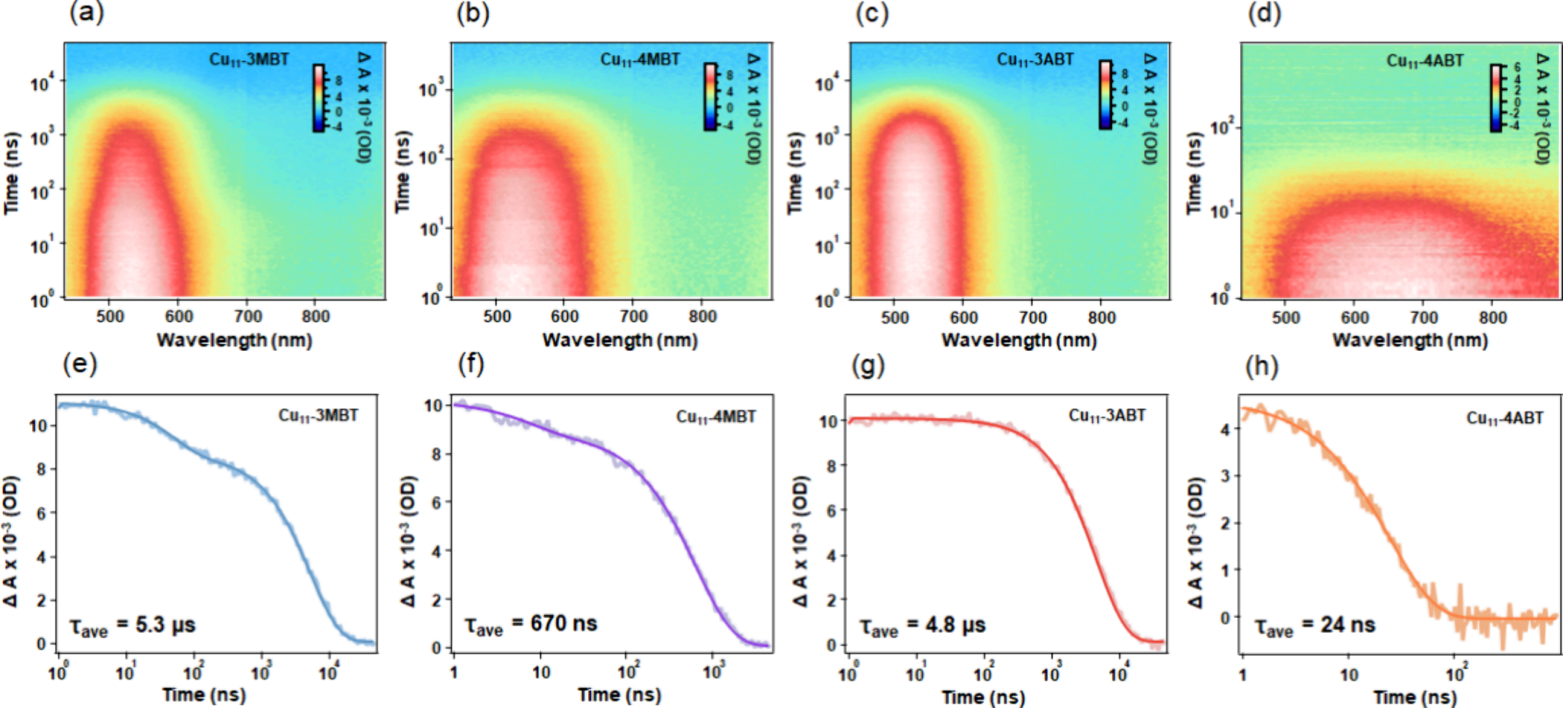
ns-TA data map at 400 nm excitation of (a) Cu_11_-3MBT, (b) Cu_11_-4MBT, (c) Cu_11_-3ABT, and (d) Cu_11_-4ABT NCs. ns-TA kinetics and fits at 541 nm for (e) Cu_11_-3MBT, (f) Cu_11_-4MBT, (g) Cu_11_-3ABT, and (h) Cu_11_-4ABT.

To further resolve the excited-state dynamics, we performed femtosecond transient absorption (fs-TA) measurements (Figure 4). Within the initial 1 ps time window, all three NCs exhibited a broad excited-state absorption (ESA) band characterized by distinct spectral features centered at approximately 530 and 650 nm. Beyond the first ps, notable spectral evolution was observed, indicating the rapid formation of a stabilized long-lived excited state, which indicates ultrafast intersystem crossing (ISC) from singlet state to triplet state. Specifically, the transient spectral profile transitions from the initially populated excited state into a more stabilized long-lived excited state, consistent with the phosphorescence-like emission behavior observed in TCSPC and ns-TA measurements (Figure S15 and Table S12). Since the Stokes shift is large (Figure 2) and ISC is ultrafast (<1 ps) in all of these four Cu NCs, the emissions observed are less likely to be thermally activated delayed fluorescence (TADF). Kinetic analysis of the 650 nm absorption band revealed a systematic trend in the decay rates, where the excited-state relaxation follows the order Cu_11_-4MBT > Cu_11_-3MBT > Cu_11_-3ABT (Figure S16 and Table S13). This faster decay in Cu_11_-4MBT suggests a more efficient nonradiative relaxation pathway, while the slower decay in the *meta*-substituted NCs—most notably Cu_11_-3ABT—indicates a more effective stabilization of the excited state, likely due to enhanced intramolecular charge transfer, ligand rigidity, and reduced vibrational relaxation. These observations collectively support that the substituent position significantly modulates the excited-state dynamics and pathway, influencing the emission lifetime and emissive behavior of the Cu_11_ NC systems.

**Figure 4:**
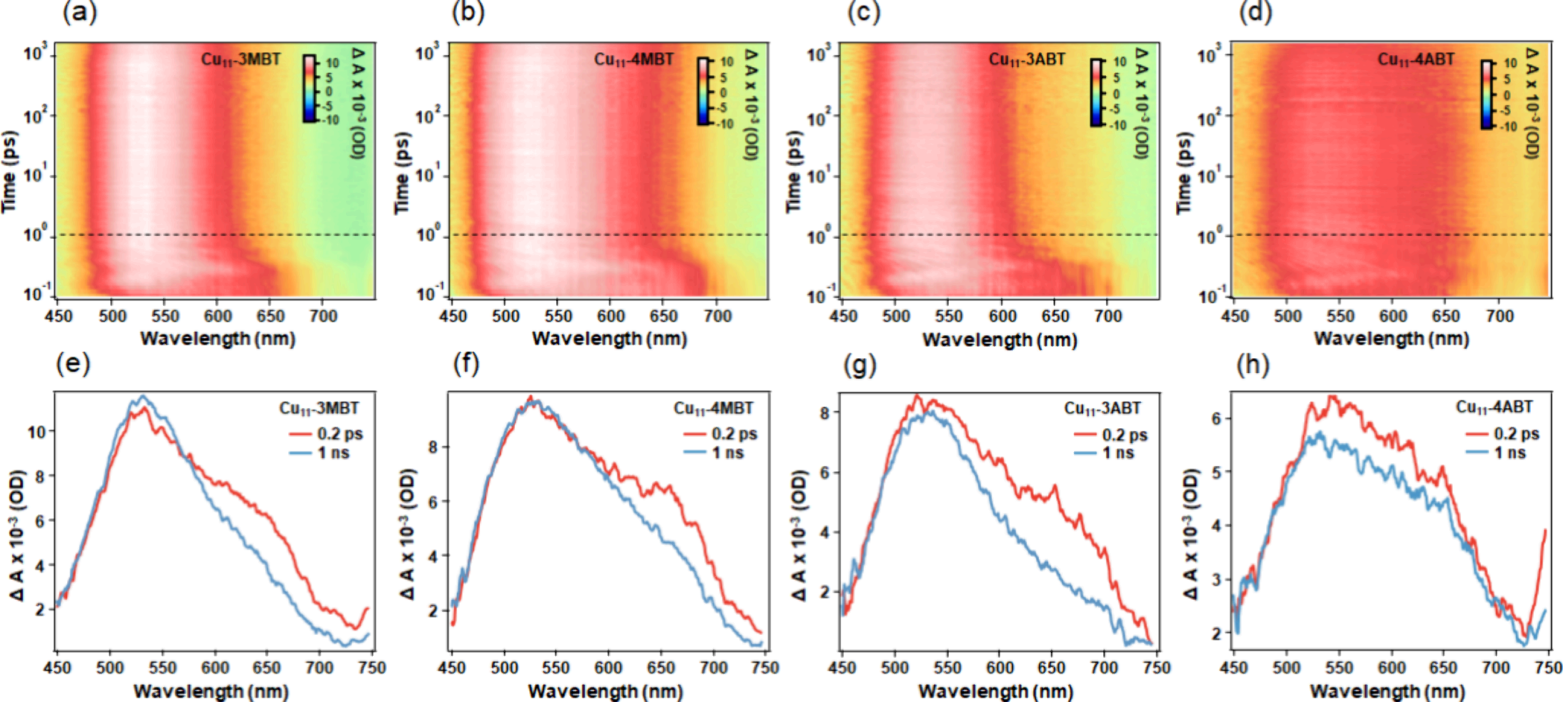
fs-TA data map at 400 nm excitation of (a) Cu_11_-3MBT, (b) Cu_11_-4MBT, (c) Cu_11_-3ABT, and (d) Cu_11_-4ABT NCs. The dashed line in each map marks the 1 ps delay time. TA spectra of (e) Cu_11_-3MBT, (f) Cu_11_-4MBT, (g) Cu_11_-3ABT, and (h) Cu_11_-4ABT NCs at delay times of 0.2 ps (red) and 1 ns (blue).

In conclusion, we report a series of Cu_11_ NC systems in which only the substitution position of the thiolate ligands is varied, enabling a clear correlation between ligand structure and photophysical properties. Long-lived triplet-like excited state involvement in the PL emission process is identified for all the Cu_11_ NCs. Among them, Cu_11_-3ABT exhibits the highest PL emission QY of 26.1% in N_2_, which is attributed to effective stabilization of the excited states, and the emission is accordingly assigned to phosphorescence.

## Supplementary Material



## Notes

Experimental section, theoretical data and other associated experimental data set (PDF)

## References

[1] Kang X., Zhu M. (2019). Tailoring the Photoluminescence of Atomically Precise Nanoclusters. Chem. Soc. Rev..

[2] Du Y., Sheng H., Astruc D., Zhu M. (2020). Atomically Precise
Noble Metal Nanoclusters as Efficient Catalysts: a Bridge between
Structure and Properties. Chem. Rev..

[3] Maity S., Kolay S., Chakraborty S., Devi A., Patra A. (2025). A Comprehensive Review
of Atomically Precise Metal Nanoclusters with
Emergent Photophysical Properties towards Diverse Applications. Chem. Soc. Rev..

[4] Liu Z.-Y., Nie Q.-B., Han B.-L., Gupta R. K., Dong G.-L., Luo G.-G., Yang Z.-L., Sun D. (2025). Atom-Precise Coinage
Metal Nanoclusters for Near-Infrared Emission: Excited-State Dynamics
and Mechanisms. Chem. Soc. Rev..

[5] Jin R., Zeng C., Zhou M., Chen Y. (2016). Atomically Precise
Colloidal Metal Nanoclusters and Nanoparticles: Fundamentals and Opportunities. Chem. Rev..

[6] Hirai H., Ito S., Takano S., Koyasu K., Tsukuda T. (2020). Ligand-Protected Gold/Silver
Superatoms: Current Status and Emerging Trends. Chem. Sci..

[7] Jin Y., Zhang C., Dong X.-Y., Zang S.-Q., Mak T. C. (2021). Shell Engineering
to Achieve Modification and Assembly of Atomically-Precise Silver
Clusters. Chem. Soc. Rev..

[8] Biswas S., Das S., Negishi Y. (2023). Progress and
Prospects in the Design of Functional
Atomically-Precise Ag (I)-Thiolate Nanoclusters and their Assembly
Approaches. Coord. Chem. Rev..

[9] Chakraborty I., Pradeep T. (2017). Atomically Precise
Clusters of Noble Metals: Emerging
Link between Atoms and Nanoparticles. Chem.
Rev..

[10] Zou X., Kang X., Zhu M. (2023). Recent Developments in the Investigation
of Driving Forces for Transforming Coinage Metal Nanoclusters. Chem. Soc. Rev..

[11] Biswas S., Das A. K., Mandal S. (2023). Surface Engineering of Atomically
Precise M­(I) Nanoclusters: From Structural Control to Room Temperature
Photoluminescence Enhancement. Acc. Chem. Res..

[12] Higaki T., Li Q., Zhou M., Zhao S., Li Y., Li S., Jin R. (2018). Toward the
Tailoring Chemistry of Metal Nanoclusters for Enhancing
Functionalities. Acc. Chem. Res..

[13] Lin H., Song X., Chai O. J. H., Yao Q., Yang H., Xie J. (2024). Photoluminescent Characterization
of Metal Nanoclusters: Basic Parameters,
Methods, and Applications. Adv. Mater..

[14] Liu Z., Sardar A., Chen S., Wang Y., Jin R. (2026). Atomically
Precise Metal Nanoclusters for Near-Infrared-II Photonics. Acc. Chem. Res..

[15] Liu Y., Yu J., Lun Y., Wang Y., Wang Y., Song S. (2023). Ligand Design
in Atomically Precise Copper Nanoclusters and Their Application in
Electrocatalytic Reactions. Adv. Funct. Mater..

[16] Zhang B., Chen J., Cao Y., Chai O. J. H., Xie J. (2021). Ligand Design
in Ligand-Protected Gold Nanoclusters. Small.

[17] Matus M. F., Häkkinen H. (2023). Understanding
Ligand-Protected Noble Metal Nanoclusters
at Work. Nat. Rev. Mater..

[18] Zhang C., Si W. D., Wang Z., Tung C. H., Sun D. (2024). Chiral Ligand-Concentration
Mediating Asymmetric Transformations of Silver Nanoclusters: NIR-II
Circularly Polarized Phosphorescence Lighting. Angew. Chem., Int. Ed..

[19] Yang T.-Q., Peng B., Shan B.-Q., Zong Y.-X., Jiang J.-G., Wu P., Zhang K. (2020). Origin of the Photoluminescence of Metal Nanoclusters:
from Metal-Centered Emission to Ligand-Centered Emission. Nanomater.

[20] Zhang Y., Zhang W., Zhang T.-S., Ge C., Tao Y., Fei W., Fan W., Zhou M., Li M.-B. (2024). Site-Recognition-Induced
Structural and Photoluminescent Evolution of the Gold–Pincer
Nanocluster. J. Am. Chem. Soc..

[21] Akiyama A., Hossain S., Biswas S., Shiraogawa T., Zhao P., Nakamoto M., Ogata D., Kawawaki T., Niihori Y., Yuasa J. (2025). Triggering
Photoluminescence
in High-Nuclear Silver Nanoclusters via Extra Silver Atom Incorporation. J. Am. Chem. Soc..

[22] Zhong Y., Zhang J., Li T., Xu W., Yao Q., Lu M., Bai X., Wu Z., Xie J., Zhang Y. (2023). Suppression
of Kernel Vibrations by Layer-by-Layer Ligand Engineering Boosts Photoluminescence
Efficiency of Gold Nanoclusters. Nat. Commun..

[23] Tian Y., Zheng W., Zhang X., Wang Y., Xiao Y., Yao D., Zhang H. (2023). Triple Ligand
Engineered Gold Nanoclusters with Enhanced
Fluorescence and Device Compatibility for Efficient Electroluminescence
Light-Emitting Diodes. Nano Lett..

[24] Zhang S.-S., Havenridge S., Zhang C., Wang Z., Feng L., Gao Z.-Y., Aikens C. M., Tung C.-H., Sun D. (2022). Sulfide Boosting
Near-Unity Photoluminescence Quantum Yield of Silver Nanocluster. J. Am. Chem. Soc..

[25] Li S., Du X.-S., Li B., Wang J.-Y., Li G.-P., Gao G.-G., Zang S.-Q. (2018). Atom-Precise Modification of Silver­(I)
Thiolate Cluster by Shell Ligand Substitution: a new Approach to Generation
of Cluster Functionality and Chirality. J. Am.
Chem. Soc..

[26] Eyyakkandy N. N., Afreen A., Vilangappurath G., Gratious S., Adarsh K., Mandal S. (2024). Modulating the Ligand Bulkiness on a Series of Au_36_(SR)_24_ Nanoclusters for Photoluminescence Enhancement. J. Phys. Chem. C.

[27] Zeng L., Wang Y., Tan J., Pei Q., Kong J., Zhang W., Ye S., Jin R., Luo Y., Zhou M. (2025). Accelerated Intersystem Crossing Enhances NIR Emission
in Au_52_(SR)_32_ Nanoclusters by Surface ligand
Engineering. Chem. Sci..

[28] Biswas S., Negishi Y. (2024). A Comprehensive Analysis
of Luminescent Crystallized
Cu Nanoclusters. J. Phys. Chem. Lett..

[29] Shi Y. e., Ma J., Feng A., Wang Z., Rogach A. L. (2021). Aggregation-Induced
Emission of Copper Nanoclusters. Aggregate.

[30] Wang Z., Chen B., Rogach A. L. (2017). Synthesis,
Optical Properties and
Applications of Light-Emitting Copper Nanoclusters. Nanoscale Horiz..

[31] Biswas S., Negishi Y. (2024). Exploring the Impact of Various Reducing Agents on
Cu Nanocluster Synthesis. Dalton Trans..

[32] Dong C., Huang R.-W., Chen C., Chen J., Nematulloev S., Guo X., Ghosh A., Alamer B., Hedhili M. N., Isimjan T. T., Han Y., Mohammed O. F., Bakr O. M. (2021). [Cu_36_H_10_(PET)_24_(PPh_3_)_6_Cl_2_] Reveals Surface
Vacancy Defects in Ligand-Stabilized Metal Nanoclusters. J. Am. Chem. Soc..

[33] Dong C., Huang R. W., Sagadevan A., Yuan P., Gutiérrez-Arzaluz L., Ghosh A., Nematulloev S., Alamer B., Mohammed O. F., Hussain I., Rueping M., Bakr O. M. (2023). Isostructural Nanocluster
Manipulation Reveals Pivotal Role of One Surface Atom in Click Chemistry. Angew. Chem., Int. Ed..

[34] Agrawal S., Shil D., Gupta A., Mukherjee S. (2024). Superstructures
of Copper Nanoclusters as NIR TADF Emitters: Solvent-dependent Optical
and Morphological Modulation. Nanoscale.

[35] Yu H., Rao B., Jiang W., Yang S., Zhu M. (2019). The Photoluminescent
Metal Nanoclusters with Atomic Precision. Coord.
Chem. Rev..

[36] Du P., Jiang W., Wei J., Li S., Shen H. (2024). Ligand Effects in Photoluminescence
of Copper Nanoclusters. Dalton Trans..

[37] Nematulloev S., Huang R. W., Yin J., Shkurenko A., Dong C., Ghosh A., Alamer B., Naphade R., Hedhili M. N., Maity P., Eddaoudi M., Mohammed O. F., Bakr O. M. (2021). [Cu_15_(PPh_3_)_6_(PET)_13_]^2+^: A Copper Nanocluster with
Crystallization
Enhanced Photoluminescence. Small.

[38] Lin X., Tang J., Zhu C., Wang L., Yang Y., Wu R. a., Fan H., Liu C., Huang J. (2023). Solvent-Mediated
Precipitating Synthesis and Optical Properties of Polyhydrido Cu_13_ Nanoclusters with Four Vertex-Sharing Tetrahedrons. Chem. Sci..

[39] Zhuo H. Y., Su H. F., Cao Z. Z., Liu W., Wang S. A., Feng L., Zhuang G. L., Lin S. C., Kurmoo M., Tung C. H. (2016). High-Nuclear Organometallic Copper­(I)–Alkynide
Clusters: Thermochromic Near-Infrared Luminescence and Solution Stability. Chem.—Eur. J..

[40] Zhang M. M., Dong X. Y., Wang Z. Y., Li H. Y., Li S. J., Zhao X., Zang S. Q. (2020). AIE Triggers the Circularly Polarized
Luminescence of Atomically Precise Enantiomeric Copper (I) Alkynyl
Clusters. Angew. Chem., Int. Ed..

[41] Jia T., Guan Z.-J., Zhang C., Zhu X.-Z., Chen Y.-X., Zhang Q., Yang Y., Sun D. (2023). Eight-Electron Superatomic
Cu_31_ Nanocluster with Chiral Kernel and NIR-II Emission. J. Am. Chem. Soc..

[42] Das A. K., Biswas S., Wani V. S., Nair A. S., Pathak B., Mandal S. (2022). [Cu_18_H_3_(S-Adm)_12_(PPh_3_)_4_Cl_2_]: Fusion of Platonic and Johnson
Solids through a Cu (0) Center and Its Photophysical Properties. Chem. Sci..

[43] Wu Z., Liu H., Li T., Liu J., Yin J., Mohammed O. F., Bakr O. M., Liu Y., Yang B., Zhang H. (2017). Contribution
of Metal Defects in the Assembly Induced Emission of Cu Nanoclusters. J. Am. Chem. Soc..

[44] Jana A., Duary S., Das A., Kini A. R., Acharya S., Machacek J., Pathak B., Base T., Pradeep T. (2024). Multicolor
Photoluminescence of Cu_14_ Clusters Modulated Using Surface
Ligands. Chem. Sci..

[45] Vilar-Vidal N., Rivas J., Lopez-Quintela M. A. (2012). Size Dependent
Catalytic Activity
of Reusable Subnanometer Copper (0) Clusters. ACS Catal..

[46] Langer R., Yadav M., Weinert B., Fenske D., Fuhr O. (2013). Luminescence
in Functionalized Copper Thiolate Clusters–Synthesis and Structural
Effects. Eur. J. Inorg. Chem..

[47] Eichhöfer A., Buth G., Lebedkin S., Kühn M., Weigend F. (2015). Luminescence in Phosphine-Stabilized
Copper Chalcogenide
Cluster Molecules- A Comparative Study. Inorg.
Chem..

[48] Li H., Zhai H., Zhou C., Song Y., Ke F., Xu W. W., Zhu M. (2020). Atomically Precise Copper Cluster
with Intensely Near-Infrared Luminescence and Its Mechanism. J. Phys. Chem. Lett..

[49] Biswas S., Shingyouchi Y., Kamiyama M., Jena M. K., Ogami M., Kawawaki T., Pathak B., Negishi Y. (2025). Deciphering
Electrocatalytic
Activity in Cu Nanoclusters: Interplay Between Structural Confinement
and Ligands Environment. Small.

[50] Frisch, M. ; Trucks, G. ; Schlegel, H. ; Scuseria, G. ; Robb, M. ; Cheeseman, J. ; Scalmani, G. ; Barone, V. ; Mennucci, B. ; Petersson, G. Gaussian 09; Gaussian, Inc.: Wallingford, CT, 2009.

[51] Stratmann R. E., Scuseria G. E., Frisch M. J. (1998). An Efficient Implementation of Time-Dependent
Density-Functional Theory for the Calculation of Excitation Energies
of Large Molecules. J. Chem. Phys..

[52] Martin R. L. (2003). Natural
Transition Orbitals. J. Chem. Phys..

