# Supplementary material for: Electronic Control of Emission Behavior in Atomically Precise Copper Nanoclusters
Source: JACS Au. 2026 Mar 31;6(4):2183–90. doi: 10.1021/jacsau.6c00121 (PMC13126154; doi:10.1021/jacsau.6c00121)
Supplement: Supplementary file 1 [file au6c00121_si_001.pdf]

Supporting Information

Electronic Control of Emission Behavior in Atomically Precise Copper Nanoclusters

Maho Kamiyama,[a†] Linlin Zeng,[b†] Milan Kumar Jena,[c,d] Yamato Shingyouchi,[e] Aoi Akiyama,[e] Tokuhisa Kawawaki,[a] Sourav Biswas,[a]* Biswarup Pathak,[c]* Meng Zhou,[b]* Yuichi Negishi[a,e]*

[a] Institute of Multidisciplinary Research for Advanced Materials, Tohoku University, Katahira 2-1-1, Aoba-ku, Sendai 980-8577, Japan.

[b] Hefei National Research Center for Physical Sciences at the Microscale, University of Science and Technology of China, Hefei, Anhui 230026, P. R. China.

[c] Department of Chemistry, Indian Institute of Technology Indore, Indore, Madhya Pradesh, 453552, India.

[d] Department of Materials Science and Metallurgical Engineering, Indian Institute of Technology Bhilai, Bhilai, Chhattisgarh, 491002, India.

[e] Department of Applied Chemistry, Tokyo University of Science,1-3 Kagurazaka, Shinjuku-ku, Tokyo 162-8601, Japan.

† Equal contributions

*Corresponding Authors

S.B.: sourav.biswas210@gmail.com

B.P.: biswarup@iiti.ac.in

M.Z.: mzhou88@ustc.edu.cn

Y.N.: negishi@rs.tus.ac.jp

Table of Contents

Name	Description	Page No.
	Experimental section	S3-S6
Table S1	Hirshfield energy calculation Cu_{11} NCs and associated S atom	S7
Table S2	Matching parameters for theoretical optimized structures	S7
Table S3	Theoretical optimized HOMO-LUMO of all four Cu_{11} NCs	S7
Table S4	Delocalization of the electron densities for Cu_{11}-3MBT NC	S8
Table S5	Delocalization of the electron densities for Cu_{11}-4MBT NC	S8
Table S6	Delocalization of the electron densities for Cu_{11}-3ABT NC	S9
Table S7	Delocalization of the electron densities for Cu_{11}-4ABT NC	S9
Table S8	NTO of Cu_{11}-3MBT NC	S9-S10
Table S9	NTO of Cu_{11}-4MBT NC	S10-S11
Table S10	NTO of Cu_{11}-3ABT NC	S11-S12
Table S11	NTO of Cu_{11}-4MBT NC	S12-S13
Table S12	Emission characteristics.	S13
Table S13	fs-TA kinetics probed at 650 nm for three Cu_{11} NCs	S13
Figure S1	XPS survey spectrum of Cu_{11} NCs	S14
Figure S2	High resolution binding energy spectrum of Cu 2p peaks of Cu_{11} NCs	S14
Figure S3	Structural architecture of the reported Cu_{11} NCs	S15
Figure S4	UV absorbance profile for all nanoclusters after 4h of air exposure	S16
Figure S5	Theoretically optimized structures of all four Cu_{11} NCs	S17
Figure S6	Simulated UV-vis absorption spectrum of all four Cu_{11} NCs	S18
Figure S7	Theoretical molecular orbital energy levels of Cu_{11}-3MBT NC	S18
Figure S8	Theoretical molecular orbital energy levels of Cu_{11}-4MBT NC	S19
Figure S9	Theoretical molecular orbital energy levels of Cu_{11}-3ABT NC	S19
Figure S10	Theoretical molecular orbital energy levels of Cu_{11}-4ABT NC	S20
Figure S11	Molecular orbital distribution with energy levels for Cu_{11}-3MBT NC	S20
Figure S12	Molecular orbital distribution with energy levels for Cu_{11}-4MBT NC	S21
Figure S13	Molecular orbital distribution with energy levels for Cu_{11}-3ABT NC	S21
Figure S14	Molecular orbital distribution with energy levels for Cu_{11}-4ABT NC	S22
Figure S15	Comparison of excited state lifetimes for all Cu_{11} NCs	S22
Figure S16	fs-TA kinetics probed at 650 nm for all Cu_{11} NCs	S23
	References	S24

Experimental Section:

Reagents

Tetrakis(acetonitrile)copper(I) tetrafluoroborate $Cu(CH_3CN)_4(BF_4)$, triphenylphosphine (PPh_3), 3-methylbenzenethiol (3-MBT), 4-methylbenzenethiol (4-MBT), 3-aminobenzenethiol (3-ABT), 4-aminobenzenethiol (4-ABT), sodium borohydride ($NaBH_4$) were procured from TCI chemicals and Borane tert-butylamine complex (BTBC) was procured from Sigma-Aldrich. HPLC grade solvents- chloroform, methanol, acetonitrile, and n-hexane were purchased from Wako Chemical.

Materials characterization

Electrospray ionization mass spectrometry (ESI-MS) was performed using a reflectron-type time of flight MS system (Bruker, microTOF II). The nanoclusters (NCs) were dissolved in a mixture of chloroform and methanol. The isotope distribution was calculated using an isotope pattern simulator (JEOL, Isotope Pattern Simulator). X-ray photoelectron spectroscopy (XPS) experiments were conducted on a JPS-9-1-MC electron spectrometer (JEOL, Tokyo, Japan) utilizing the Mg-Kα line (1253.6 eV) as the excitation source. All the binding energies were referenced to the neutral C 1s peak at 284.6 eV. UV-Vis absorption spectra were acquired using a JASCO V-770 spectrophotometer. The time-resolved fluorescence decay profiles were recorded by a time-correlated single photon counting spectrometer (FluoTime 300, PicoQuant, Germany), excited at 375 nm picosecond laser (PDL 820, PicoQuant diode laser). The PLQYs were measured using an absolute method with an integrating sphere method on a HAMAMATSU C11347 Quantaurus-QY spectrometer. Therefore, no reference dye was used.

Synthesis of Cu_{11}–3MBT NC

The entire reaction process was carried out in a 50 mL vial at ambient condition. First, commercially available ($Cu(CH_3CN)_4BF_4$) (160 mg, 0.509 mmol) was dissolved by adding acetonitrile (10 mL). Then, 130 mg of PPh_3 was added to the reaction mixture and kept stirring until dissolving PPh_3 for 10 minutes. After fully dissolving PPh_3 powder in solution, 47μL of 3-MBT and kept stirring until dissolving PPh_3 for 30 minutes. Then, 200 mg of BTBT (in 3

mL of methanol) and 3 mL of acetone was quickly added in order. The color of the solution immediately changed from clear to orange. The reaction mixture was stirred for 3 hours. Subsequently, the solvent was removed with a rotary evaporator, and the residue was dissolved in 10 mL of methanol. The mouth of the vial was sealed and allowed to stand at dark place for 1 day to obtain the Cu_{11}-3MBT as orange colored, crystals.

Synthesis of Cu_{11}–4MBT NC

The entire reaction process was carried out in a 50 mL vial at ambient condition as reported in our previous result.[S1] First, commercially available ($Cu(CH_3CN)_4BF_4$) (160 mg, 0.509 mmol) was dissolved by adding acetonitrile (10 mL). Then, 130 mg of PPh_3 was added to the reaction mixture and kept stirring until dissolving PPh_3 for 10 minutes. After fully dissolving PPh_3 powder in solution, 36.8 mg of 4-MBT (in 200 μL of acetonitrile) and kept stirring until dissolving PPh_3 for 30 minutes. Then, 200 mg of Borane tert-butylamine complex (in 3 mL of methanol) and 3 mL of acetone was quickly added in order. The color of the solution immediately changed from clear to orange. The reaction mixture was stirred for 3 hours. Subsequently, the solvent was removed with a rotary evaporator, and the residue was dissolved in 10 mL of methanol. The mouth of the vial was sealed and allowed to stand at dark place for 1 day to obtain the Cu_{11}-4MBT, as orange colored, crystals.

Synthesis of Cu_{11}–3ABT NC

The entire reaction process was carried out in a 50 mL vial at ambient condition as per our previously reported method.[S1] First, commercially available ($Cu(CH_3CN)_4BF_4$), 160 mg, 0.509 mmol was dissolved by adding acetonitrile (4 mL) and chloroform (4 mL) in that order. Then, 130 mg of PPh_3 was added to the reaction mixture and kept stirring until dissolving PPh_3 for 10 minutes. After fully dissolving PPh_3 powder in solution, 20 μL of 3-ABT was added. The color of the solution changed from clear to yellow. After confirming that the color no longer changes, 30 mg of $NaBH_4$ (in 3 mL of methanol) was quickly added. The color of the solution immediately changed from yellow to reddish brown. The reaction mixture was stirred for 3 hours. Subsequently, the solvent was removed with a rotary evaporator, and the residue was dissolved in 10 mL of methanol. The mouth of the vial was sealed and allowed to stand at dark place for 2 days to obtain the Cu_{11}-3ABT, as yellowish brown colored, crystals.

Synthesis of Cu_{11}–4ABT NC

The entire reaction process was carried out in a 50 mL vial in an ice bath. First, commercially available ($Cu(CH_3CN)_4BF_4$), 160 mg, 0.509 mmol was dissolved by adding acetonitrile (4 mL) and chloroform (4 mL) in that order. Then, 130 mg of PPh_3 was added to the reaction mixture and kept stirring until dissolving PPh_3 for 10 minutes. After fully dissolving PPh_3 powder in solution, 46.3 mg of 4-ABT (in 200 μL of acetonitrile) was added. The color of the solution changed from clear to yellow. After confirming that the color no longer changes, 30 mg of $NaBH_4$ (in 3 mL of methanol) was quickly added. The color of the solution immediately changed from yellow to reddish brown. The reaction mixture was stirred for 3 hours. Subsequently, the solvent was removed with a rotary evaporator, and the residue was dissolved in 10 mL of methanol. The mouth of the vial was sealed and allowed to stand at dark place for 5 days to obtain the Cu_{11}-4ABT, as red colored, crystals.

Femtosecond and Nanosecond Transient Absorption Spectra.

Femtosecond TA measurements were performed on a commercial Ti:Sapphire laser (Coherent Astrella, 800 nm, 35 fs, 1.2 mJ, 5 KHz). The pump pulse was generated using a commercial optical parametric amplifier (TOPAS-prime, LightConverison). The probe pulse was produced by focusing a small portion of the laser fundamental into a sapphire plate. The femtosecond pump-probe measurements were performed on a home-built setup. The white light and the pump beam were overlapped into the sample and the linear polarizations were set at the magic angle (54.7°) to record the isotropic response. The instrument response function of whole device is around 80 fs. The concentration of samples in different solvents was adjusted to an absorbance around 0.3 OD at excitation wavelength in a 1 mm quartz cuvette. No degradation was observed throughout the experiment that checked by steady absorption spectra. The ns-TA spectra were measured by a commercial spectrometer (NANO100, Time-Tech Spectra). The generation of the pump beam is the same as that in fs-TA. The probe beam was generated from a supercontinuum laser (LEUKOS-DISCO, French) with the spectral region from 350 nm to 1800 nm, the repetition rate is 2 kHz, pulse width is 700 ps - 1 ns. There is no photodegrading after ns-TA experiments by checking the steady-state absorption spectra.

Methods of Density Functional Theory (DFT) calculations

The electronic structure calculation of the four considered NCs, Cu_{11}-3MBT, Cu_{11}-4MBT, Cu_{11}-3ABT, and Cu_{11}-4ABT, was performed using the density functional theory (DFT) method as implemented in the Gaussian 09 package.[S2] For non-metal atoms (S, P, N, C, and H), the Becke's three-parameter hybrid exchange functional combined with Lee–YangParr's (B3LYP) correlation functional was employed together with the 6-31G* basis set. The metal (Cu) atoms were treated with the LANL2DZ effective core potential (ECP).[S3-6] The optical absorption spectra were calculated using time-dependent density functional theory (TD-DFT) method as available in the Gaussian 09 package.[S2-S7] To gain a better understanding of the electronic structure of the NCs, the Kohn-Sham orbital analysis was also performed. Frontier molecular orbital and delocalisation of the electron densities among core, shell and the other ligands of each NCs were calculated using the Multiwfn package (version 3.8), which provided valuable insights into Cu metal and ligand orbital contributions.[S8] The molecular orbital energy levels of all four NCs are plotted using Chemcraft software. The molecular orbital energy level diagrams for all four nanoclusters were visualised using Chemcraft software. Natural transition orbital (NTO) analysis was performed to obtain the dominant hole–particle representation of key electronic transitions in NCs.[S9] Further, the Hirshfeld charge density analysis was carried out on the optimised geometries to quantify the charge distribution at the sulfur coordination sites.[S10]

Table S1. Hirshfeld energy calculation Cu_{11} NCs and associated S atom.

System	Total Hirshfeld	Hirshfeld ⟨qS⟩
Cu_{11}-3MBT	-1.456	-0.1621
Cu_{11}-4MBT	−1.476	-0.16397
Cu_{11}-3ABT	−1.418	-0.1532
Cu_{11}-4ABT	−1.42	-0.1578

Table S2. Matching parameters for theoretical optimized structures.

System	Parameters	Crystal Data	Theoretical Optimized Structure
Cu_{11}-4MBT	Average Cu-Cu distance	2.8331 ±0.0175	2.7687 ±0.0070
	Average Cu-S distance	2.2703 ±0.0090	2.2443 ±0.0012
	Average Cu-P distance	2.2142 ±0.0045	2.2240
	Average < Cu-Cu-S	109.77 ±2.383	112.23 ±4.539
	Average < S-Cu-P	127.145 ±5.759	129.64 ±3.892
Cu_{11}-3ABT	Average Cu-Cu distance	2.8383 ±0.0133	2.7214 ±0.0042
	Average Cu-S distance	2.2506 ±0.0105	2.2421 ±0.0098
	Average Cu-P distance	2.205 ±0.0017	2.2242
	Average < Cu-Cu-S	108.72 ±1.775	110.72 ±2.445
	Average < S-Cu-P	129.315 ±11.6	130.428 ±5.782

Table S3. Theoretical optimized HOMO-LUMO of all four Cu_{11} NCs.

NC	HOMO (eV)/State	LUMO (eV)/State	Gap (eV)
Cu_{11}-3MBT	-4.79 eV/451	-1.21 eV /452	3.57 eV
Cu_{11}-4MBT	-5.00 eV/451	-1.58 eV/452	3.42 eV
Cu_{11}-3ABT	-4.38 eV/451	-0.89 eV/452	3.48 eV
Cu_{11}-4ABT	-4.66 eV/451	-1.41 eV/452	3.24 eV

Table S4. Delocalization of the electron densities among core, shell and the other ligands of Cu_{11}-3MBT NC.

State	Core (Cu_5)	Shell (Cu)	Ligands
442	14.96	16.36	68.66
443	31.32	15.81	52.85
444	38.11	21.13	40.75
445	35.02	14.49	50.48
449	31.27	20.17	48.54
450	39.95	17.16	42.88
451	47.18	8.50	44.30
452	27.68	31.67	40.64
453	14.84	46.48	38.66
454	24.83	20.64	54.52
455	25.79	18.63	55.57
463	2.63	9.95	87.40

Table S5. Delocalization of the electron densities among core, shell and the other ligands of Cu_{11}-4MBT NC.

State	Core (Cu_5)	Shell	Ligand
447	48.81	12.75	38.43
437	33.08	32.70	34.20
440	45.35	20.41	34.23
449	49.44	8.77	41.78
441	40.34	18.07	41.57
442	50.06	16.39	33.53
443	51.45	18.77	29.77
445	42.01	18.50	39.48
450	50.84	16.60	32.54
451	51.02	16.44	32.52
452	27.20	15.59	57.19
453	10.98	16.74	72.26
454	28.70	4.44	66.84
455	21.67	43.81	34.50
456	10.75	17.50	7174
457	10.53	16.54	72.92
458	2.26	1.08	96.65
459	7.15	20.60	72.23

Table S6. Delocalization of the electron densities among core, shell and the other ligands of Cu_{11}-3ABT NC.

State	Core (Cu_5)	Shell	Ligand
446	31.79	11.35	46.84
447	46.33	9.35	44.30
448	21.77	9.26	68.95
449	25.13	11.17	63.68
451	39.51	22.76	37.71
452	24.69	33.5	41.34
453	17.44	47.41	35.13
454	24.53	27.32	48.14
461	10.53	32.34	57.29

Table S7. Delocalization of the electron densities among core, shell and the other ligands of Cu_{11}-4ABT NC.

State	Core (Cu_5)	Shell	Ligand
433	42.62	27.19	30.18
434	42.76	26.30	30.92
435	40.34	33.69	25.95
443	54.12	12.48	33.93
444	46.20	9.29	43.64
445	50.08	12.11	37.54
446	50.19	11.88	37.33
447	47.85	10.60	40.63
449	48.22	14.13	37.13
450	47.38	12.64	39.13
452	26.77	16.37	57.45
453	27.55	5.07	57.36
454	27.67	4.96	67.35
455	17.96	40.16	41.86
462	20.47	23.98	55.53

Table S8. NTOs of Cu_{11}-3MBT NC.

n	λ (nm)	Major transitions
1	425.3	H→L (1.00)
2	403.2	H-1→L (0.92), H-2→L (0.05)
3	399.8	H-2→L (0.75), H-3→L (0.25)
4	398.9	H-3→L (0.72), H-2→L (0.21), H-1→L (0.07)
5	374.0	H→L+1 (0.96)

6	370.6	H-4→L (0.64), H-5→L (0.25), H-6→L (0.07)
7	368.0	H-5→L (0.71), H-4→L (0.15), H-6→L (0.14)
8	360.6	H→L+2 (0.60), H-6→L (0.26)
9	358.3	H-1→L+1 (0.45), H-2→L+1 (0.23), H-7→L (0.12), H-6→L (0.07), H→L+2 (0.06)
10	356.8	H-1→L+1 (0.22), H-6→L (0.19), H-8→L (0.14), H→L+2 (0.11), H-7→L (0.11), H-2→L+1 (0.08), H-4→L (0.08), H-9→L (0.07)
11	355.6	H-2→L+1 (0.56), H-1→L+1 (0.31), H→L+2 (0.06)
12	353.5	H-7→L (0.48), H-2→L+1 (0.14), H-6→L (0.11), H→L+2 (0.10), H-3→L+1 (0.07)
14	346.3	H-9→L (0.45), H-3→L+1 (0.17), H-8→L (0.13), H-6→L (0.12), H-7→L (0.12)
18	336.5	H-8→L (0.43), H-9→L (0.19), H-10→L (0.15), H-1→L+2 (0.11), H-7→L (0.09)
19	331.1	H-10→L (0.78), H-8→L (0.10), H-11→L (0.07)
23	325.9	H-5→L+1 (0.59), H-4→L+1 (0.34)
25	320.0	H-11→L (0.57), H-7→L+1 (0.17), H-6→L+1 (0.15), H-10→L (0.05)
33	311.0	H-3→L+3 (0.31), H→L+7 (0.15), H-9→L+1 (0.14), H-8→L+1 (0.11), H-4→L+2 (0.09), H→L+8 (0.07), H-5→L+2 (0.06)
35	308.8	H-1→L+3 (0.41), H-6→L+2 (0.15), H→L+10 (0.14), H-1→L+11 (0.11), H-1→L+4 (0.09), H→L+11 (0.05), H→L+9 (0.05)
38	306.8	H-6→L+2 (0.63), H→L+9 (0.14), H→L+8 (0.12), H→L+10 (0.06), H-5→L+2 (0.06)
39	306.3	H-2→L+3 (0.55), H-2→L+11 (0.14), H-2→L+4 (0.12), H-2→L+6 (0.06), H-3→L+3 (0.05)
41	305.2	H→L+11 (0.24), H-12→L (0.22), H-7→L+2 (0.14), H→L+12 (0.11), H-1→L+9 (0.10), H-1→L+4 (0.05)
45	303.4	H→L+11 (0.32), H-12→L (0.22), H-2→L+3 (0.11), H-1→L+7 (0.07), H-1→L+4 (0.06), H-1→L+9 (0.06)
50	301.6	H-3→L+5 (0.16), H-2→L+6 (0.15), H-10→L+1 (0.13), H-2→L+7 (0.12), H-1→L+7 (0.11), H-3→L+6 (0.10), H→L+11 (0.06), H-1→L+9 (0.06)
51	301.2	H-3→L+5 (0.35), H→L+11 (0.16), H→L+12 (0.09), H→L+14 (0.09), H-1→L+7 (0.08), H-12→L (0.06), H-3→L+6 (0.06)

Table S9. NTOs of Cu_{11}-4MBT NC.

n	λ (nm)	Major transitions
1	453.0	H→L (0.97)
2	452.6	H-1→L (0.94)
3	450.9	H-2→L (0.97)
4	449.9	H-3→L (1.00)
5	412.6	H-4→L (1.00)
6	393.9	H-6→L (0.63), H-5→L (0.34)
7	393.9	H-5→L (0.63), H-6→L (0.33)
8	385.2	H-9→L (0.74), H-10→L (0.23)
9	384.9	H-10→L (0.74), H-9→L (0.22)

10	378.7	H-7→L (0.80), H-8→L (0.11)
11	375.2	H-8→L (0.73), H-7→L (0.10)
21	346.1	H-4→L+1 (1.00)
22	345.5	H-4→L+2 (1.00)
26	330.6	H-6→L+2 (0.47), H-5→L+1 (0.39), H-5→L+2 (0.08), H-6→L+1 (0.06)
28	320.7	H-9→L+1 (0.35), H-10→L+2 (0.31), H-10→L+1 (0.11), H-9→L+2 (0.11), H-7→L+1 (0.07)
29	320.6	H-9→L+2 (0.38), H-10→L+1 (0.33), H-9→L+1 (0.14), H-10→L+2 (0.11)
34	313.9	H-10→L+2 (0.42), H-9→L+1 (0.30), H-14→L (0.17), H-13→L (0.10)
35	313.2	H-14→L (0.81), H-10→L+2 (0.09), H-9→L+1 (0.07)
36	312.9	H-8→L+1 (0.55), H-12→L (0.17), H-8→L+2 (0.15), H-13→L (0.13)
37	312.7	H-8→L+2 (0.70), H-13→L (0.20), H-8→L+1 (0.07)
38	308.1	H-2→L+5 (0.57), H→L+5 (0.29), H-2→L+8 (0.11)
39	307.5	H-3→L+5 (0.74), H-1→L+5 (0.14), H-3→L+8 (0.13)
40	305.6	H→L+5 (0.20), H→L+8 (0.18), H→L+12 (0.18), H-2→L+5 (0.12), H-2→L+12 (0.07), H→L+9 (0.07), H-1→L+5 (0.06)
41	305.4	H-1→L+5 (0.25), H-1→L+12 (0.23), H-1→L+8 (0.23), H-1→L+9 (0.09), H-3→L+5 (0.07)
43	304.2	H→L+4 (0.32), H-2→L+4 (0.22), H-2→L+13 (0.08), H-1→L+4 (0.07), H-1→L+9 (0.07)
44	304.1	H-3→L+3 (0.26), H-1→L+3 (0.18), H-2→L+4 (0.13), H-1→L+9 (0.08), H→L+3 (0.08), H-1→L+4 (0.07), H-2→L+3 (0.06)
49	302.6	H-1→L+6 (0.37), H→L+7 (0.26), H-2→L+7 (0.10), H→L+3 (0.08), H-1→L+4 (0.07), H-17→L (0.05)
53	300.9	H-1→L+6 (0.21), H-2→L+7 (0.13), H-11→L+1 (0.12), H→L+7 (0.10), H-2→L+6 (0.06), H→L+11 (0.06)
54	300.8	H-1→L+7 (0.17), H→L+6 (0.15), H-2→L+6 (0.11), H-11→L+1 (0.11), H-11→L+2 (0.07), H-3→L+7 (0.07), H-1→L+11 (0.06), H→L+10 (0.06), H-2→L+7 (0.05)
57	300.0	H-1→L+7 (0.15), H→L+6 (0.08), H-2→L+10 (0.08), H-3→L+11 (0.08), H-1→L+3 (0.06), H-1→L+9 (0.06), H-2→L+14 (0.06), H-3→L+3 (0.06), H-1→L+10 (0.06), H-3→L+13 (0.05)

Table S10. NTOs of Cu_{11}-3ABT NC.

**n**	**λ (nm)**	**Major transitions**
1	424.7	H→L (1.00)
2	401.9	H-1→L (0.89), H-2→L (0.08)
3	399.2	H-2→L (0.86), H-3→L (0.08), H-1→L (0.06)
4	398.2	H-3→L (0.90), H-2→L (0.05)
5	374.3	H→L+1 (0.96)
6	371.2	H-4→L (0.46), H-5→L (0.38), H-6→L (0.12)
7	367.9	H-6→L (0.42), H-5→L (0.41), H-4→L (0.10), H-7→L (0.07)

8	361.2	H→L+2 (0.55), H-6→L (0.15), H-4→L (0.12), H-7→L (0.06), H-5→L (0.06)
9	358.4	H-1→L+1 (0.20), H-2→L+1 (0.18), H-7→L (0.18), H-4→L (0.11), H→L+2 (0.11), H-6→L (0.09), H-8→L (0.07), H-10→L (0.06)
10	357.0	H-2→L+1 (0.37), H-1→L+1 (0.20), H→L+2 (0.13), H-4→L (0.10), H-6→L (0.06), H-8→L (0.06)
11	355.8	H-1→L+1 (0.49), H-7→L (0.18), H-2→L+1 (0.16), H→L+2 (0.08)
12	354.7	H-2→L+1 (0.30), H-7→L (0.29), H-1→L+1 (0.12), H-5→L (0.11), H→L+2 (0.07)
14	346.9	H-10→L (0.43), H-3→L+1 (0.28), H-6→L (0.12), H-7→L (0.10)
18	338.0	H-8→L (0.43), H-1→L+2 (0.17), H-10→L (0.14), H-7→L (0.09), H-9→L (0.08)
19	332.0	H-9→L (0.73), H-12→L (0.14), H-8→L (0.09)
25	320.7	H-12→L (0.44), H-7→L+1 (0.27), H-6→L+1 (0.15), H-9→L (0.09)
36	308.9	H-6→L+2 (0.36), H→L+8 (0.20), H→L+10 (0.13), H→L+9 (0.10), H-5→L+2 (0.08)
37	308.4	H-5→L+2 (0.35), H-3→L+4 (0.25), H-3→L+3 (0.10), H-1→L+3 (0.10), H-2→L+3 (0.06)
38	307.3	H-6→L+2 (0.22), H-1→L+3 (0.17), H-3→L+4 (0.15), H→L+7 (0.11), H-3→L+3 (0.09), H-1→L+4 (0.07), H-1→L+11 (0.06)
40	306.4	H-2→L+3 (0.46), H-2→L+4 (0.17), H-2→L+11 (0.15), H-15→L (0.06)
41	306.0	H-13→L (0.24), H-15→L (0.18), H→L+9 (0.16), H-16→L (0.15), H-2→L+3 (0.10), H-14→L (0.07)
42	305.6	H-7→L+2 (0.45), H-8→L+1 (0.11), H-10→L+1 (0.10), H-1→L+9 (0.10), H-3→L+3 (0.08), H→L+11 (0.07), H-3→L+4 (0.06)
48	302.3	H-9→L+1 (0.39), H-2→L+4 (0.14), H-12→L+1 (0.11), H→L+11 (0.09), H-2→L+3 (0.07), H-2→L+6 (0.07), H-2→L+9 (0.05)
49	301.7	H-3→L+5 (0.39), H-3→L+6 (0.15), H-9→L+1 (0.11), H-3→L+4 (0.08), H→L+12 (0.07), H→L+13 (0.07), H-1→L+10 (0.06)

Table S11. NTOs of Cu_{11}-4ABT NC.

n	λ (nm)	Major transitions
1	472.7	H→L (0.74), H-2→L (0.14), H-3→L (0.07)
2	471.9	H-1→L (0.61), H-2→L (0.21), H-3→L (0.10), H→L (0.08)
3	469.1	H-2→L (0.67), H-1→L (0.27), H→L (0.06)
4	468.2	H-3→L (0.85), H→L (0.10), H-1→L (0.06)
5	427.5	H-4→L (0.97)
6	418.8	H-5→L (0.97)
7	418.1	H-6→L (0.97)
8	406.6	H-7→L (0.91), H-8→L (0.09)
9	400.6	H-8→L (0.74), H-9→L (0.13), H-7→L (0.10)
22	356.2	H-4→L+1 (0.77), H-6→L+1 (0.10), H-5→L+2 (0.06)
23	355.6	H-4→L+2 (0.65), H-6→L+2 (0.18), H-5→L+1 (0.10)
26	348.9	H-6→L+2 (0.42), H-5→L+1 (0.26), H-5→L+2 (0.17), H-6→L+1 (0.15)
27	340.4	H-8→L+1 (0.52), H-7→L+1 (0.33), H-4→L+1 (0.08)
32	335.4	H-13→L (0.42), H-7→L+2 (0.38), H-12→L (0.10), H-8→L+2 (0.09)

49	317.3	H-2→L+3 (0.36), H→L+10 (0.14), H-9→L+2 (0.11), H-1→L+9 (0.10), H-3→L+5 (0.08), H-15→L (0.05), H-3→L+4 (0.05)
50	317.2	H-3→L+3 (0.37), H-1→L+10 (0.14), H→L+9 (0.07), H-2→L+5 (0.05), H-9→L+1 (0.05), H-9→L+2 (0.05)
51	316.8	H-16→L (0.88), H→L+5 (0.06)
58	308.4	H-17→L (1.00)
59	308.3	H-18→L (1.00)
61	305.8	H→L+11 (0.46), H-1→L+12 (0.21), H-2→L+14 (0.10), H→L+14 (0.06)
65	304.2	H-2→L+11 (0.24), H-2→L+12 (0.20), H→L+13 (0.17), H-3→L+12 (0.08), H-2→L+8 (0.06), H→L+14 (0.06), H-2→L+16 (0.05), H-1→L+12 (0.05)
67	303.8	H-1→L+14 (0.26), H-3→L+11 (0.25), H-3→L+12 (0.16), H-3→L+8 (0.08), H-1→L+13 (0.06), H-3→L+9 (0.05), H-2→L+12 (0.05)
72	300.1	H→L+8 (0.15), H-4→L+3 (0.14), H-2→L+4 (0.13), H→L+12 (0.11), H→L+10 (0.10), H-2→L+8 (0.09), H-2→L+11 (0.08), H-2→L+13 (0.06), H-3→L+12 (0.06)

Table S12. Emission characteristics.

**Samples**	PL QY (%)		TCSPC Lifetime		ns-TA Lifetime in air
	Air	N_2	Air	N_2	
Cu_{11}-3MBT	8.2	14	4.6 μs	5.0 μs	5. 3 μs
Cu_{11}-4MBT	1.8	2.3	701 ns	772 ns	670 ns
Cu_{11}-3ABT	11.6	26.1	5.8 μs	10.0 μs	4.8 μs
Cu_{11}-4ABT	0.3	0.3	-	-	24 ns

Table S13. fs-TA kinetics probed at 650 nm for three Cu_{11} NCs.

**Samples**	**Fast decay (ps)**
Cu_{11}-3MBT	0.37
Cu_{11}-4MBT	0.25
Cu_{11}-3ABT	0.47

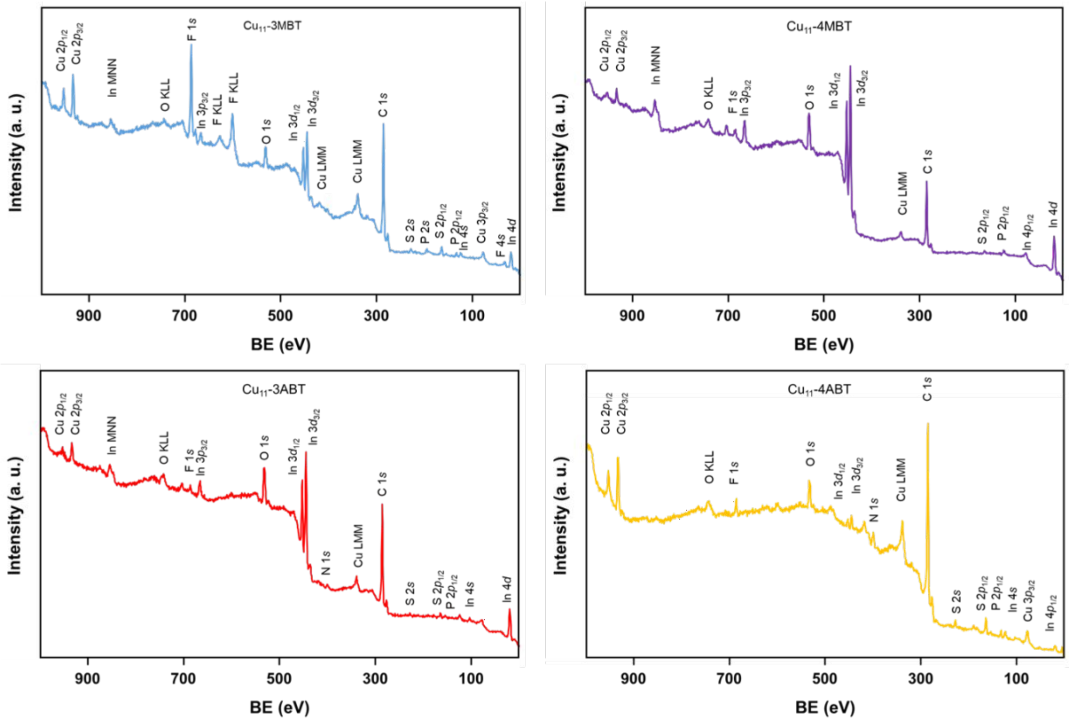

Figure S1. XPS survey spectrum of all four Cu_{11} NCs.

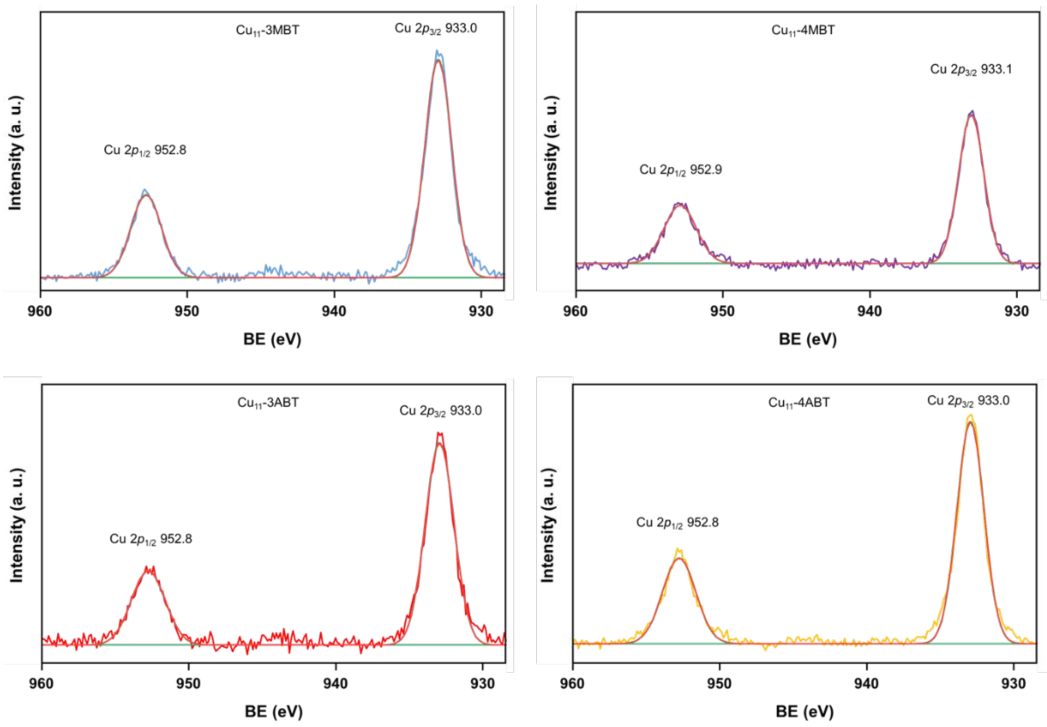

Figure S2. High resolution binding energy spectrum of Cu 2p peaks of all four Cu_{11} NCs. This suggests presence of Cu(I) species in all of these samples.

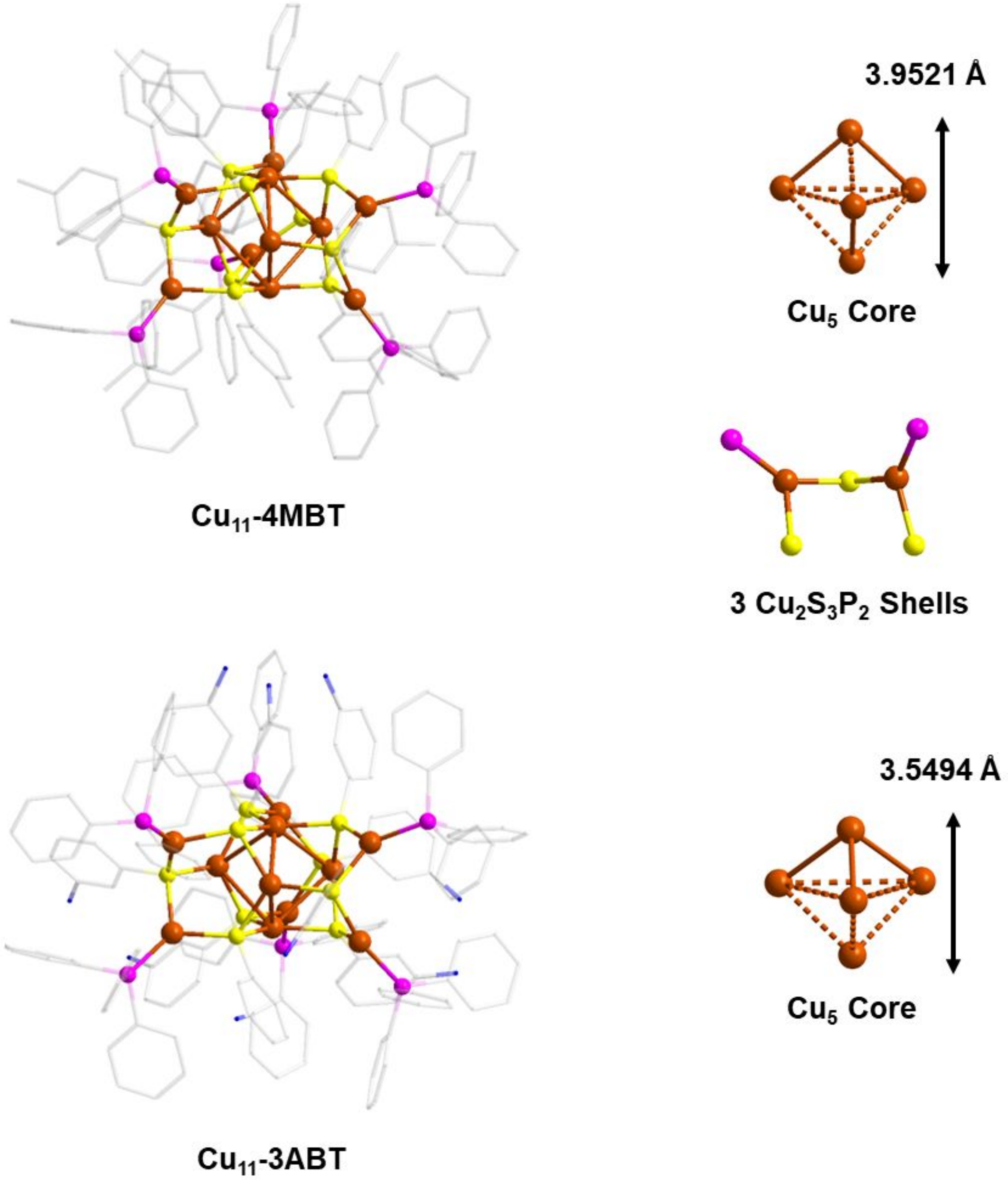

Figure S3. Structural architecture of the reported Cu_{11}-4MBT and Cu_{11}-3ABT NCs. This figure is redrawn from the reference S1.

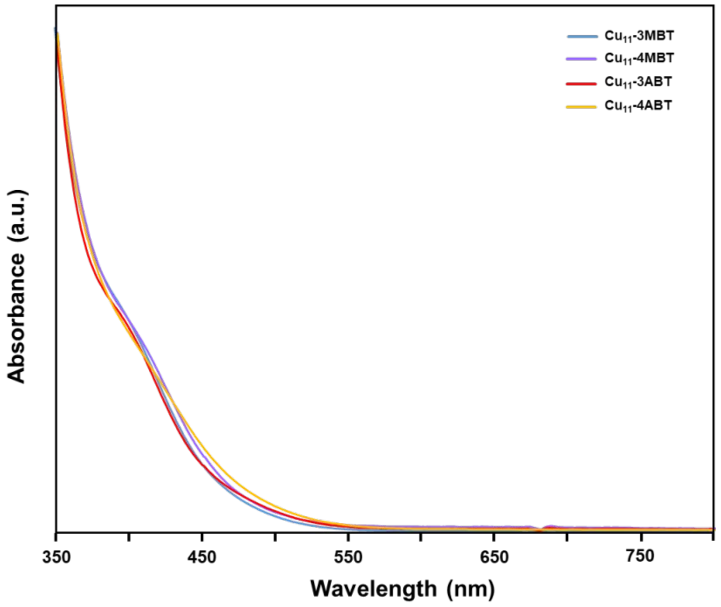

Figure S4. UV absorbance profile for all nanoclusters after 4h of air exposure.

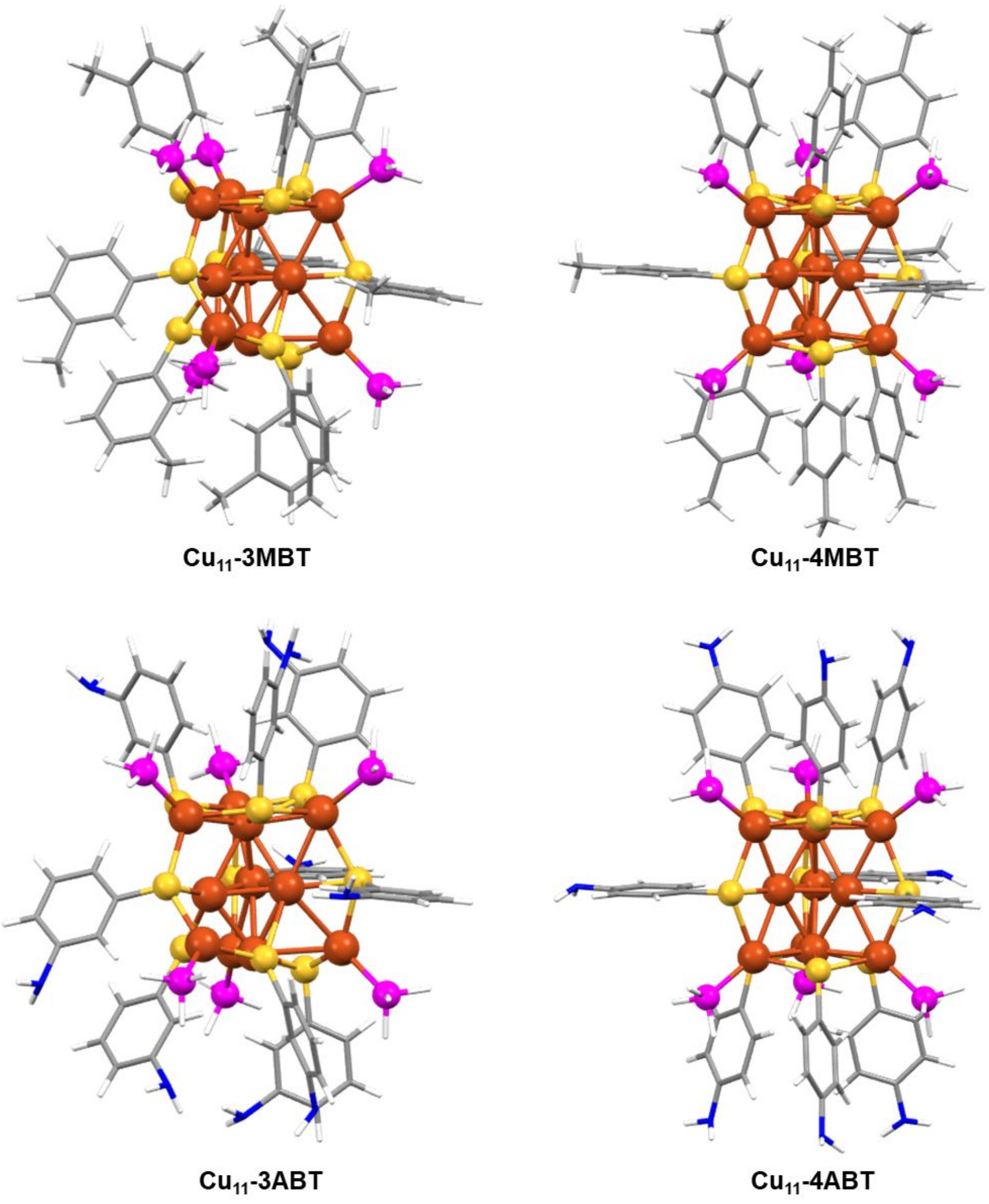

Figure S5. Theoretically optimized structures of all four Cu_{11} NCs.

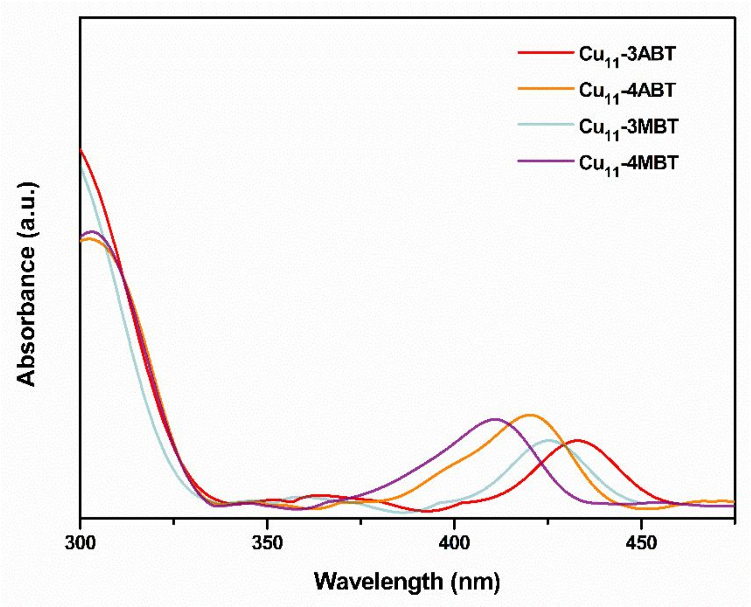

Figure S6. Simulated UV-vis absorption spectrum of all four Cu_{11} NCs.

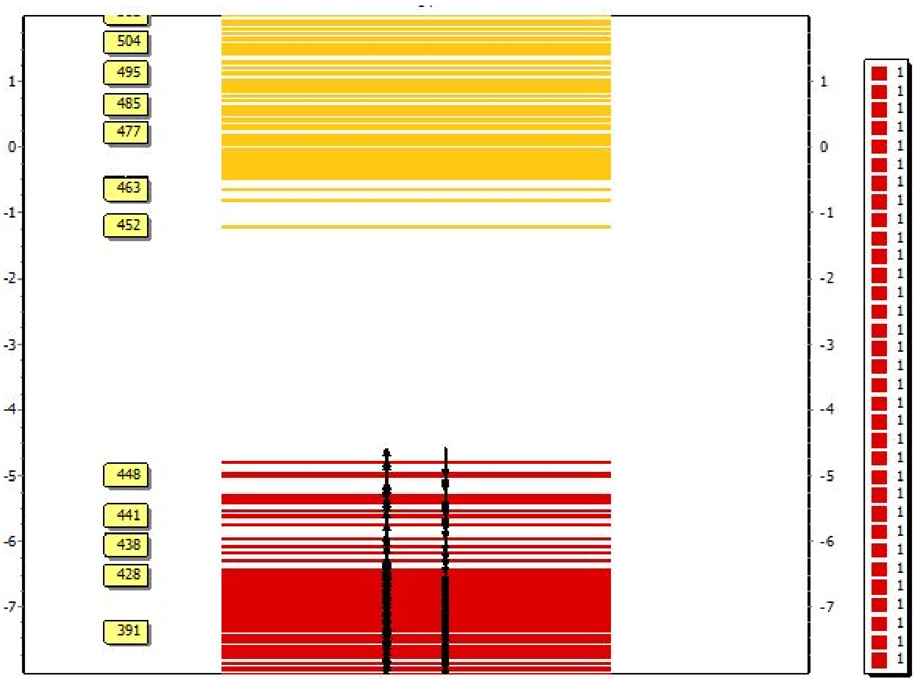

Figure S7. Theoretical molecular orbital energy levels of Cu_{11}-3MBT NC.

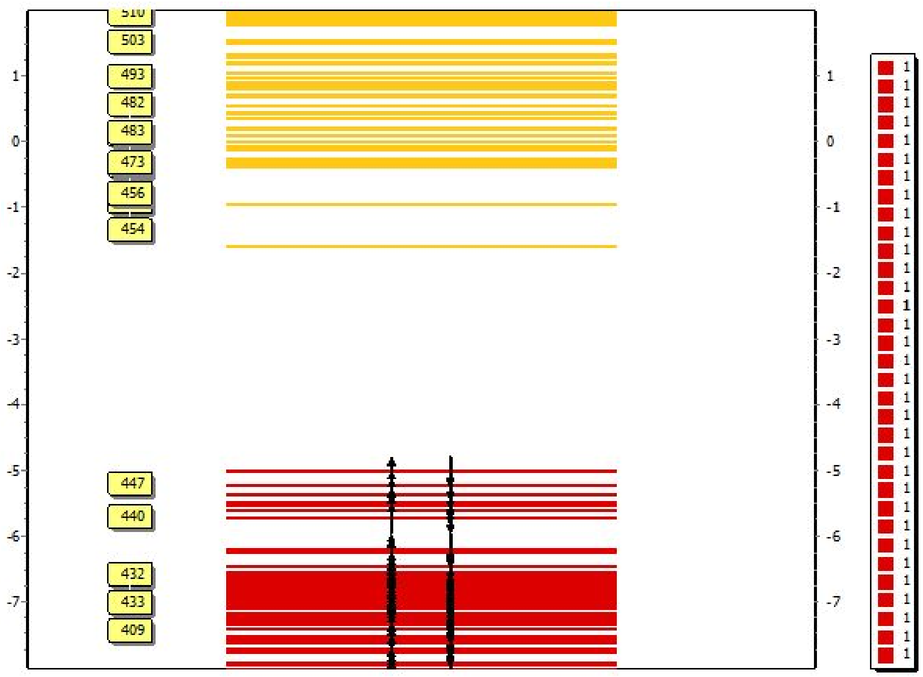

Figure S8. Theoretical molecular orbital energy levels of Cu_{11}-4MBT NC.

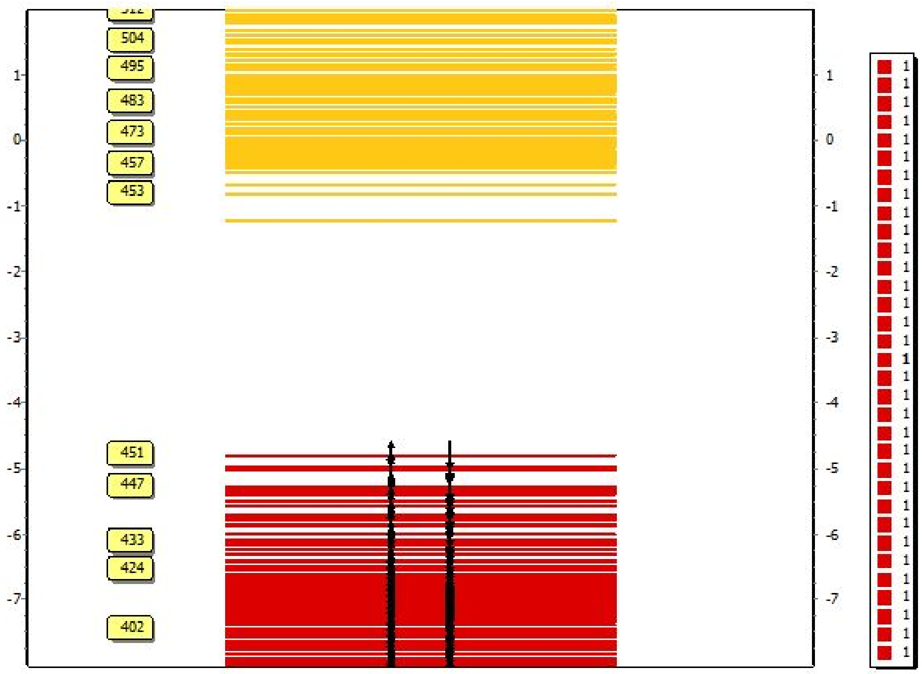

Figure S9. Theoretical molecular orbital energy levels of Cu_{11}-3ABT NC.

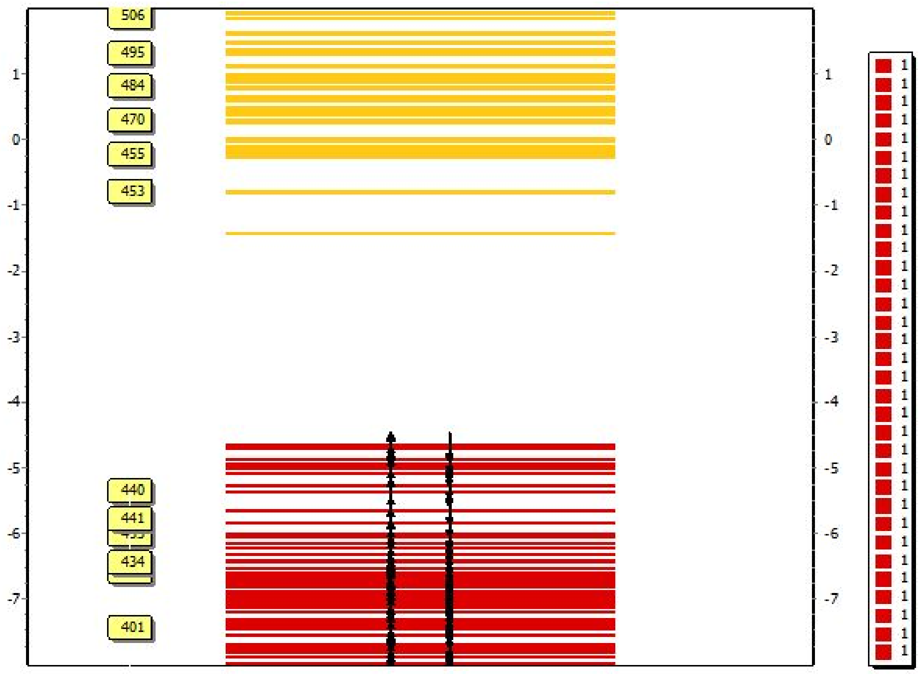

Figure S10. Theoretical molecular orbital energy levels of Cu_{11}-4ABT NC.

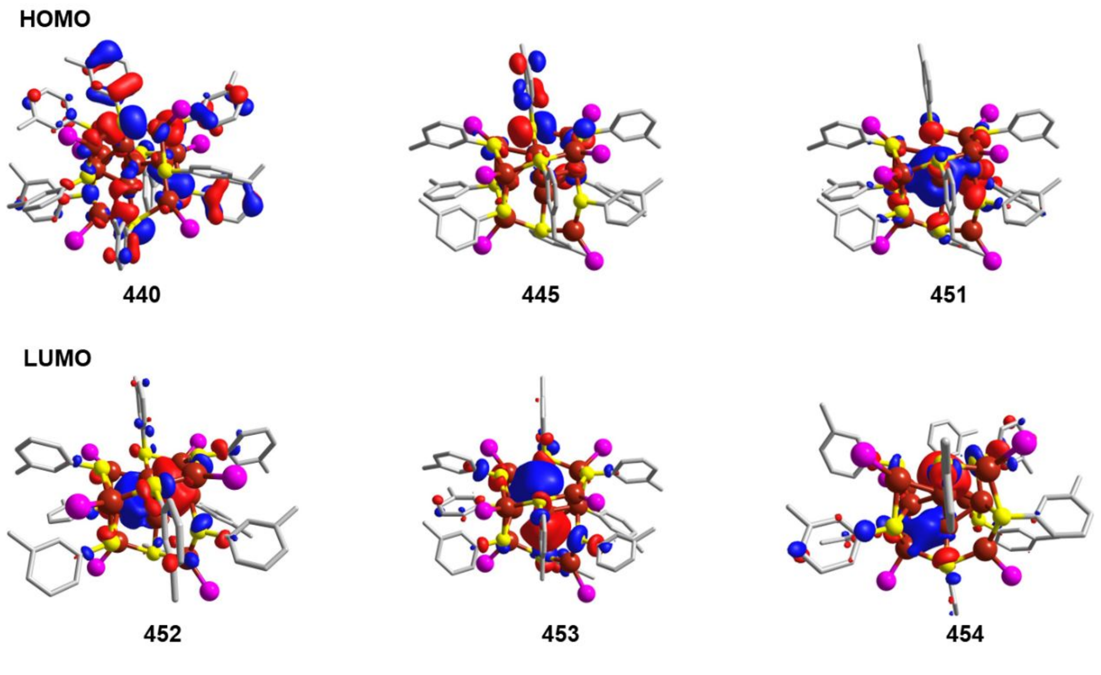

Figure S11. Molecular orbital distribution with energy levels for Cu_{11}-3MBT NC.

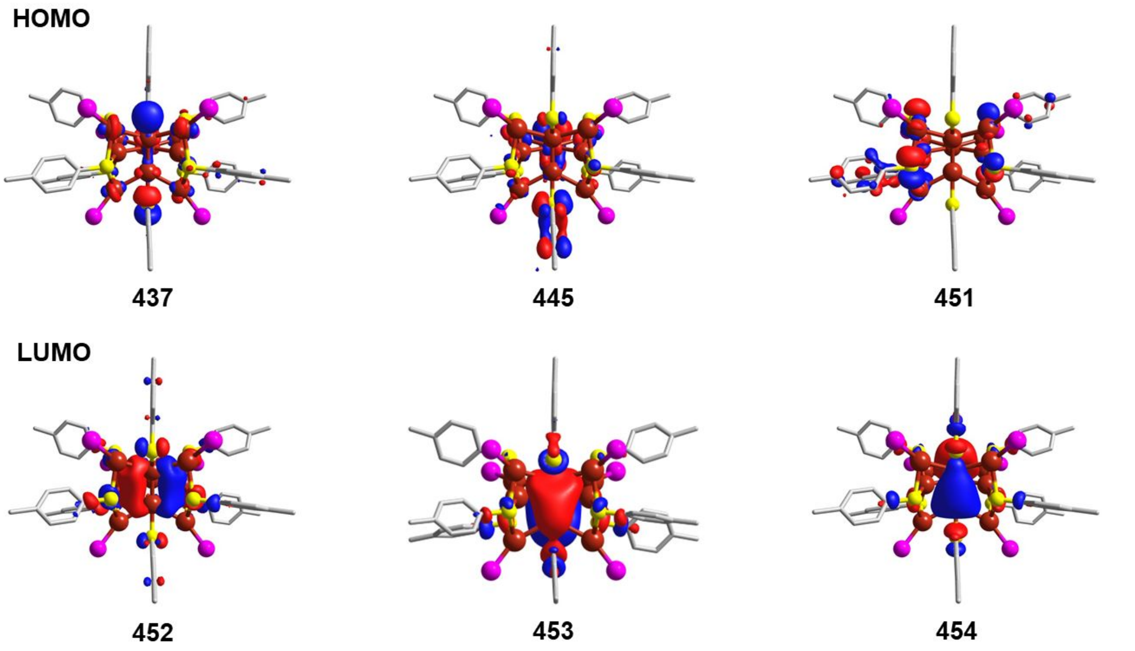

Figure S12. Molecular orbital distribution with energy levels for Cu_{11}-4MBT NC.

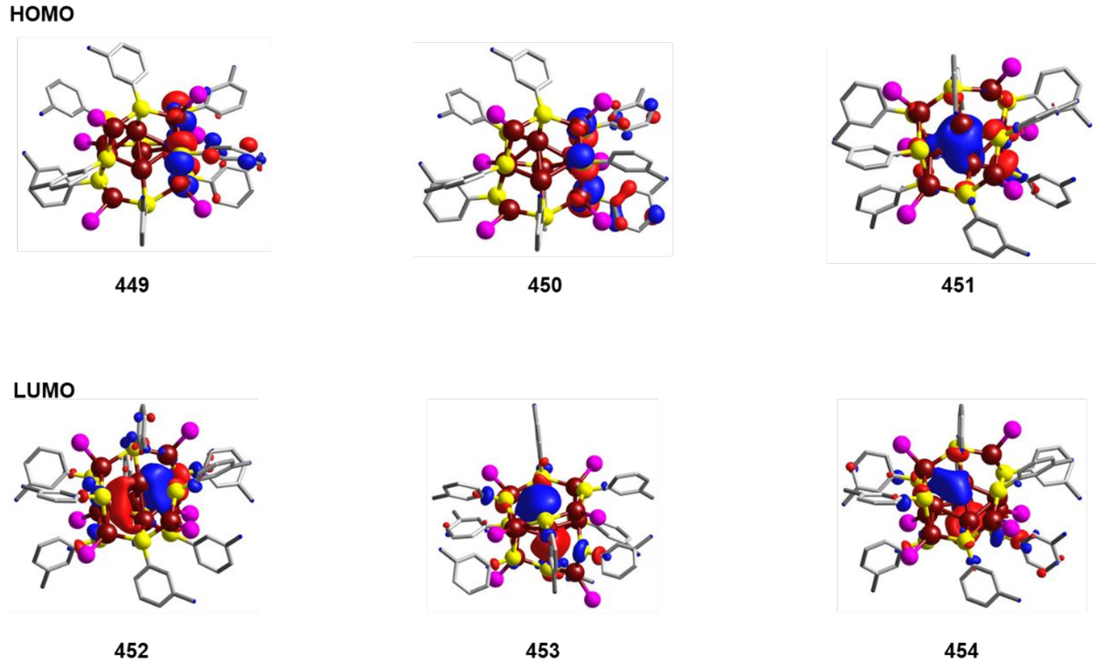

Figure S13 Molecular orbital distribution with energy levels for Cu_{11}-3ABT NC.

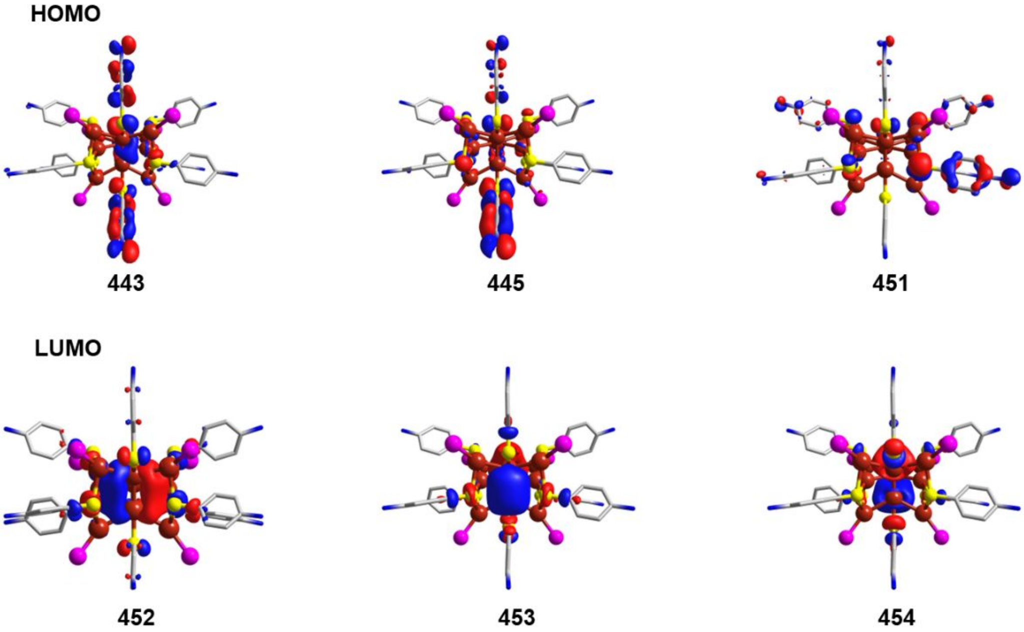

Figure S14. Molecular orbital distribution with energy levels for Cu_{11}-4ABT NC.

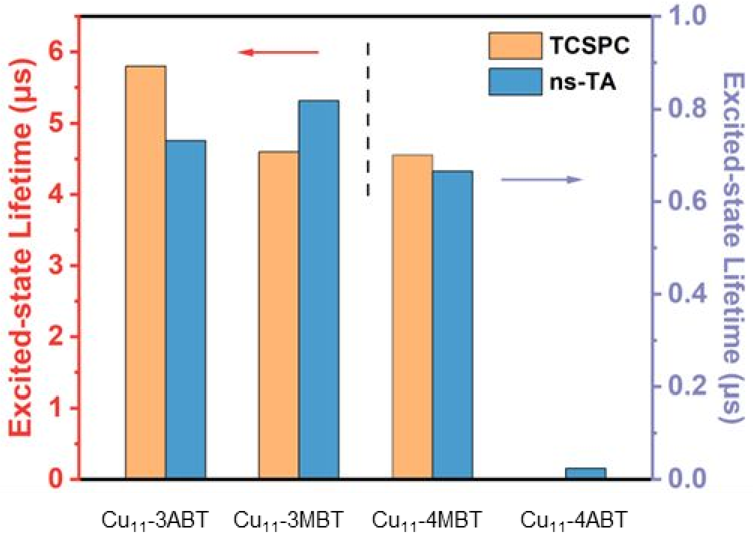

Figure S15. Comparison of excited state lifetimes for all Cu_{11} NCs.

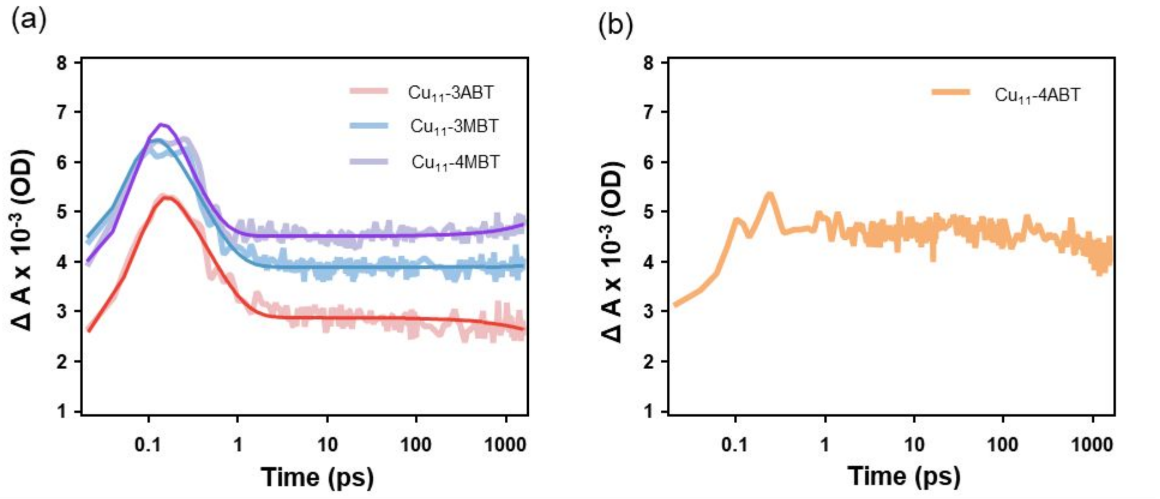

Figure S16. fs-TA kinetics probed at 650 nm for (a) three Cu_{11} NCs and (b) Cu_{11}-4ABT NC.

References

S1. Biswas, S.; Shingyouchi, Y.; Kamiyama, M.; Jena, M. K.; Ogami, M.; Kawawaki, T.; Pathak, B.; Negishi, Y. Deciphering Electrocatalytic Activity in Cu Nanoclusters: Interplay Between Structural Confinement and Ligands Environment. *Small* **2025**, *21*, 2500302.

S2. Frisch, M.; Trucks, G.; Schlegel, H.; Scuseria, G.; Robb, M.; Cheeseman, J.; Scalmani, G.; Barone, V.; Mennucci, B.; Petersson, G. Gaussian Inc. Wallingford Ct 2009, 2009.

S3. Becke, A. D. Density - Functional Thermochemistry. I. The Effect of the Exchange-only Gradient Correction. J. Chem. Phys. 1992, 96, 2155-2160.

S4. McLean, A.; Chandler, G. Contracted Gaussian Basis Sets for Molecular Calculations. I. Second Row Atoms, Z= 11–18. J. Chem. Phys. 1980, 72, 5639-5648.

S5. Hay, P. J.; Wadt, W. R. Ab Initio Effective Core Potentials for Molecular Calculations. Potentials for the Transition Metal Atoms Sc to Hg. J. Chem. Phys. 1985, 82, 270-283.

S6. Hay, P. J.; Wadt, W. R. Ab Initio Effective Core Potentials for Molecular Calculations. Potentials for K to Au Including the Outermost Core Orbitals. J. Chem. Phys. 1985, 82, 299-310.

S7. Stratmann, R. E.; Scuseria, G. E.; Frisch, M. J. An Efficient Implementation of Time-Dependent Density-Functional Theory for the Calculation of Excitation Energies of Large Molecules. J. Chem. Phys. 1998, 109, 8218– 8224.

S8. Lu, T.; CHEN, F.-W. Calculation of Molecular Orbital Composition. Acta Chim. Sinica 2011, 69, 2393.

S9. Martin, R. L. Natural Transition Orbitals. J. Chem. Phys. 2003, 118, 4775–4777.

S10. Hirshfeld, F. L. Bonded-Atom Fragments for Describing Molecular Charge Densities Theoret. Claim. Acta (Berl.) **1977**, 44, 129– 138.
